# Synthesis and Properties of Cobalt/Nickel-Iron-Antimony(III, V)-Oxo Tartrate Cluster-Based Compounds

**DOI:** 10.3390/molecules29030591

**Published:** 2024-01-25

**Authors:** Weiyang Wen, Yanqi Wang, Tianyu Pan, Qianqian Hu, Huiping Xiao, Nannan Wang, Xiaoqi Li, Xinxiong Li, Bing Hu, Xiaoying Huang

**Affiliations:** 1 State Key Laboratory of Structural Chemistry, Fujian Institute of Research on the Structure of Matter, Chinese Academy of Sciences, Fuzhou 350002, China; wenweiyang@fjirsm.ac.cn (W.W.); pantianyu@fjirsm.ac.cn (T.P.); huqianqian@fjirsm.ac.cn (Q.H.); 2 College of Chemistry, Fuzhou University, Fuzhou 350108, China; hpxiao@163.com (H.X.); lxx@fzu.edu.cn (X.L.); 3 Fujian Provincial Key Laboratory of Ecology-Toxicological Effects & Control for Emerging Contaminants, College of Environmental and Biological Engineering, Putian University, Putian 351100, China; wangyanqi2023@outlook.com; 4 University of Chinese Academy of Sciences, Beijing 100049, China; 5 Fujian Provincial Key Laboratory of Advanced Materials Oriented Chemical Engineering, College of Chemistry and Materials Science, Fujian Normal University, Fuzhou 350007, China; 13140521491@163.com (N.W.); qiyiguo202203@163.com (X.L.)

**Keywords:** mixed-valence, antimony-oxo cluster, transition metal, photodegradation, proton conduction, magnetism

## Abstract

Two types of isostructural iron-cobalt/nickel-antimony-oxo tartrate cluster-based compounds, namely (H_3_O)(Me_2_NH_2_)[*M*(H_2_O)_6_]_2_[Fe^II^_2_Sb^III^_12_(*μ*_4_-O)_3_(*μ*_3_-O)_8_(tta)_6_]·6H_2_O (*M* = Co (**1**); Ni (**3**)), H_5/3_[Co_2.5_Fe^II^_4/3_Fe^III^_3_(H_2_O)_13_Sb^V^_1/3_Fe^III^_2/3_(*μ*_4_-O)_2_(*μ*_3_-O)_4_Sb^III^_6_(*μ*_3_-O)_2_(tta)_6_]·2H_2_O (**2**) and H_2_[Ni_2.25_Fe^II^_1.5_Fe^III^_3_(H_2_O)_14_Sb^V^_0.25_Fe^III^_0.75_(*μ*_4_-O)_2_(*μ*_3_-O)_4_Sb^III^_6_(*μ*_3_-O)_2_(tta)_6_]·2H_2_O (**4**) (H_4_tta = tartaric acid) were synthesized via simple solvothermal reactions. All the clusters in the structures adopt sandwich configurations, that is, bilayer sandwich configuration in **1** and **3** and monolayer sandwich configuration in **2** and **4**. Interestingly, the monolayer sandwiched compounds **2** and **4** represent rare examples of cluster-based compounds containing mixed-valence Sb(III, V), whose center of the intermediate layer is the co-occupied [Fe*_x_*Sb^V^_1−*x*_]. This is different from that of previously reported sandwich-type antimony-oxo clusters in which the center position is either occupied by a transition metal ion or a Sb(V) alone. Thus, the discovery of title compounds **2** and **4** makes the evolution of center metal ion more complete, that is, from *M*, *M_x_*Sb^V^_1−*x*_ to Sb^V^. All the title compounds were fully characterized, and the photocatalysis, proton conduction and magnetism of compounds **2** and **4** were studied.

## 1. Introduction

In recent years, antimony oxides have been used in a wide range of applications such as nonlinear optics and catalysis [1,2,3,4,5,6]. Crystalline Sb(III)-oxo-cluster-based compounds possess a precise structure and thus their performance can be effectively optimized through function-oriented structural design from the atomic point of view [1,2]. The design and synthesis of organic–inorganic hybrid compounds based on Sb(III)-oxo clusters have drawn increasing attention recently. The organic components may play two roles in these hybrids, that is, acting as charge-balancing cations or as ligands entering or linking the Sb(III)-oxo-based clusters [7,8,9,10,11,12,13,14,15]. In the synthesis of these hybrids, potassium antimony tartrate has been widely favored by scholars for its feature of undergoing decomposition and rearrangement in water to form {Sb_3_(*μ*_3_-O)(tta)_3_} scaffolds with three bridging oxygen groups, which further capture other metal ions to form a cluster-based structure [13,16].

To date, potassium antimony tartrate has been extensively combined with transition metal ions to form a range of transition-metal-antimony-oxo tartrate cluster-based structures with different structural types. In term of properties, however, mostly only the magnetic behavior has been investigated as shown in Table S1 [13,14,15,16,17,18,19,20,21]. From the structure point of view, the clusters adopt either sandwich or bowl configurations. The sandwich configuration is dominant, and it can be further subdivided into bilayer sandwiches and monolayer sandwiches depending on the intermediate layer. In bilayer-sandwiched compounds, the intermediate portion is occupied by the {*M*Sb_3_}_2_ (*M* = Co, Ni, Cu, Ni or Cr) layer (e.g., in Na_4_[Cu_2_Sb_12_(*µ*_3_-O)_6_(*µ*_3_-OH)_2_(*µ*_4_-O)_3_(tta)_6_]·19H_2_O) [14,16,18]. By contrast, the intermediate layer of monolayer ones is generally occupied by {*M*O_6_*M’*_3_*M’’_n_*} (*M* = V, Mn, Fe, Cu, Sb(V); *M’* = Mn, Fe, Co, Cu; *M’’* = Mn, Fe, Co, Cu, Cd), where *M* is the center metal ion, *M’* are the three metal ions surrounding *M* and *M’’* are the peripheral metal ions [13,14,15,16,17,19,20,21]. Notably, the vast majority of the center metal ions *M* are a transition metal ion (e.g., in (H_4_TPOM)[Fe^III^_4_Mn_3_Sb_6_(*μ*_4_-O)_6_(*μ*_3_-O)_2_(L-tta)_6_(H_2_O)]·16H_2_O, TPOM = tetrakis(4-pyridyl-oxymethylene)-methane) [17], while it is rarely occupied by Sb(V) (e.g., in H_5_{*A*Cd(H_2_O)_6_[*A*(H_2_O)_3_Co_3_Sb^V^Sb^III^_6_(*μ*_3_-O)_8_(L-tta)_6_]}·7H_2_O (*A* = Cd_0.5_ + Co_0.5_)) [19,21]. However, the transformation process of the center metal from transition metal ions to Sb(V) ions is still ambiguous. Therefore, it is interesting and meaningful to explore the transformation process of the central metal in the monolayer sandwich-typed transition-metal-antimony-oxo tartrate clusters which may provide a hint to design new transition-metal-antimony-oxo clusters of this type.

Herein, we report four transition-metal-antimony-oxo tartrate cluster-based compounds, namely (H_3_O)(Me_2_NH_2_)[*M*(H_2_O)_6_]_2_[Fe^II^_2_Sb^III^_12_(*μ*_4_-O)_3_(*μ*_3_-O)_8_(tta)_6_]·6H_2_O (*M* = Co (**1**); Ni (**3**)), H_5/3_[Co_2.5_Fe^II^_4/3_Fe^III^_3_(H_2_O)_13_Sb^V^_1/3_Fe^III^_2/3_(*μ*_4_-O)_2_(*μ*_3_-O)_4_Sb^III^_6_(*μ*_3_-O)_2_(tta)_6_]·2H_2_O (**2**) and H_2_[Ni_2.25_Fe^II^_1.5_Fe^III^_3_(H_2_O)_14_Sb^V^_0.25_Fe^III^_0.75_(*μ*_4_-O)_2_(*μ*_3_-O)_4_Sb^III^_6_(*μ*_3_-O)_2_(tta)_6_]·2H_2_O (**4**). Compounds **2** and **4** are isostructural featuring a monolayer sandwich-typed–cluster-based structure while **1** and **3** are also isostructural characterized by a bilayer sandwich-typed cluster. Interestingly, **2** and **1** can be crystallized as the major phase and minor phase, respectively, from one single reaction. A similar phenomenon occurs for the synthesis of **4** and **2**. It is noteworthy that **2** and **4** represent the rare examples of cluster-based compounds containing mixed valence states of antimony (III and V), and the [*M*O_6_] polyhedron at the center of the intermediate layer of the cluster is co-occupied by Fe(III) and Sb(V), making the evolution of the central metal of monolayer–sandwich-typed transition-metal-antimony-oxo clusters clearer and more intuitive. Selected properties of compounds **2** and **4** have been investigated, including proton conductivity, photocatalysis and magnetism. The results indicate that the proton conduction rates are 3.86 × 10^−4^ S·cm^−1^ for **2** and 2.12 × 10^−4^ S·cm^−1^ for **4**, respectively. The photocatalytic degradation of methylene blue (MB) solutions could reach 93.58% for **2** and 98.30% for **4** after 8 h at 25 °C, respectively. In addition, magnetic analyses show that the antiferromagnetic interaction is dominant both in **2** and **4**.

## 2. Results and Discussion

### 2.1. Discussion of Synthesis Methods

**Figure 1:**
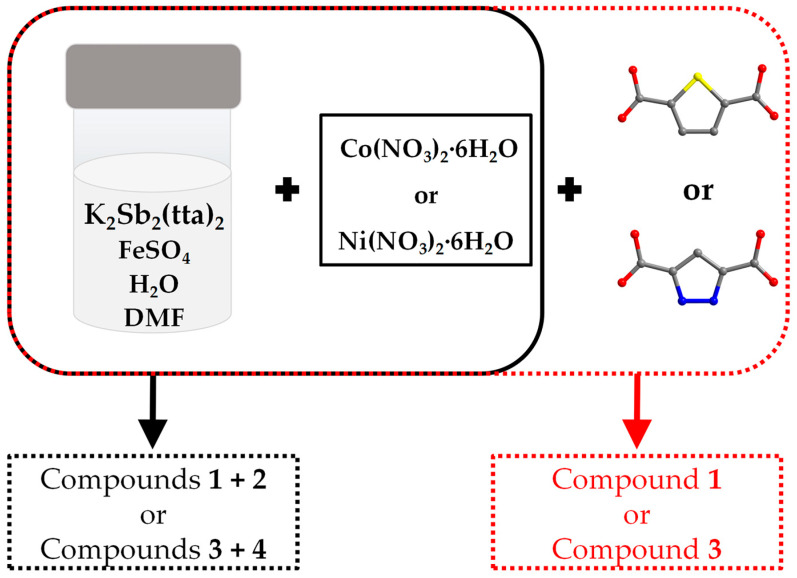
Schematic illustration of the synthesis of the title compounds. Reaction conditions as follows: heated at 100 °C for 4 days. Color scheme: S, yellow; N, blue; O, red and C, gray. For clarity, the hydrogen atoms are omitted.

Figure 1 illustrates the synthesis methods for compounds **1**, **2**, **3** and **4**. When only Co(NO_3_)_2_·6H_2_O or Ni(NO_3_)_2_·6H_2_O is added to the reaction vessel, compounds **1** and **2** or **3** and **4** can be obtained together. However, when 0.05 g of thiophene-2,5-dicarboxylic acid or pyrazole-3,5-dicarboxylic acid was added with Co(NO_3_)_2_·6H_2_O or Ni(NO_3_)_2_·6H_2_O at the same time as the starting materials of two syntheses, it was found that compound **2** as well as compound **4** could not be obtained, and only a small amount of compounds **1** and **3** could be obtained, respectively, and the quality of the crystals deteriorated compared to that from the original reaction. But when the reaction solvents were changed from 3 mL H_2_O as well as 1 mL DMF to 4 mL H_2_O, all of the above-mentioned crystals could not be obtained. In summary, we speculate that compounds **2** and **4** are synthesized under sensitive conditions and that minor pH changes may affect their synthesis [14].

### 2.2. Description of the Structures

**Figure 2:**
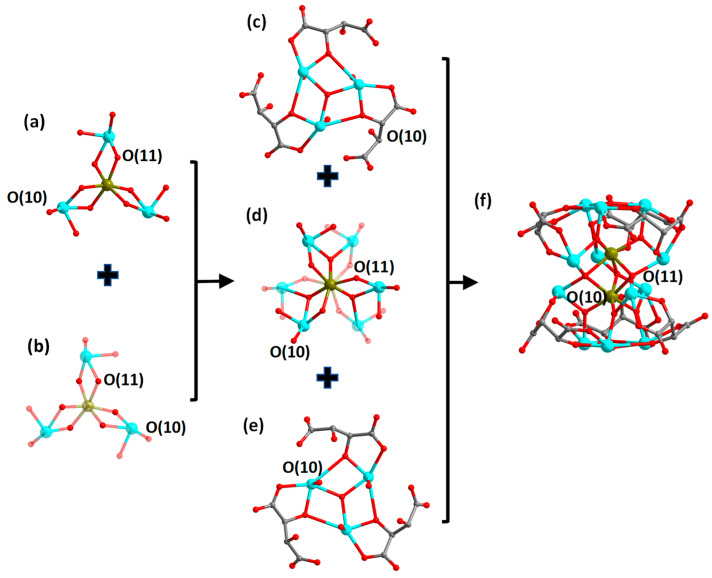
Structural evolution of the anion cluster [Fe^II^_2_Sb^III^_12_(*μ*_3_-O)_8_(*μ*_4_-O)_3_(tta)_6_]^6−^ in compound **1**. Upper (**a**) and lower (**b**) structure of the {Fe(*μ*_3_-O)_3_(*μ*_4_-O)_3_Sb_3_} layer. For clarity, the transparency of some atoms in the substructure as well as atomic bonds is set to 70%. Structure of the top layer {Sb^III^_3_(*μ*_3_-O)(tta)_3_} (**c**), the intermediate layer {Fe_2_(*μ*_3_-O)_6_(*μ*_4_-O)_3_Sb_6_} (**d**), the bottom layer {Sb^III^_3_(*μ*_3_-O)(tta)_3_} (**e**) and the anionic cluster {Fe_2_Sb_12_(*μ*_3_-O)_8_(*μ*_4_-O)_3_(tta)_6_} (**f**). Color scheme: Fe, brown; Sb, turquoise; O, red and C, gray. Hydrogen atoms are omitted for clarity.

Single-crystal structure analysis indicates that compounds **1** and **3** are isostructural; so are compounds **2** and **4**. Therefore, we chose compounds **1** and **2** as examples for the structural descriptions. The compound (H_3_O)(Me_2_NH_2_)[Co(H_2_O)_6_]_2_[Fe^II^_2_Sb^III^_12_(*μ*_4_-O)_3_(*μ*_3_-O)_8_(tta)_6_]·6H_2_O (**1**) crystallizes in the hexagonal crystal system, *P*6_3_22 space group; its asymmetric unit comprises one-third of the molecular unit, i.e., one-third of the anionic cluster [Fe^II^_2_Sb^III^_12_(*μ*_3_-O)_8_(*μ*_4_-O)_3_(tta)_6_]^6−^, two-thirds of the cationic [Co(H_2_O)_6_]^2+^ complexes, one-third of H_3_O^+^ cations and one-third of [Me_2_NH_2_]^+^ cations, as well as two lattice water molecules. Its anionic cluster portion [Fe^II^_2_Sb^III^_12_(*μ*_3_-O)_8_(*μ*_4_-O)_3_(tta)_6_]^6−^ presents a bilayer sandwich configuration. The top and bottom layers of the cluster are two {Sb^III^_3_(*μ*_3_-O)(tta)_3_} scaffolds (Figure 2c,e). The middle part is in a bilayer sandwich configuration consisting of two {Fe^II^(*μ*_3_-O)_3_(*μ*_4_-O)_3_Sb^III^_3_} layers sharing three *μ*_4_-O(11) atoms (Figure 2a,b,d). The center of the {Fe^II^(*μ*_3_-O)_3_(*μ*_4_-O)_3_Sb^III^_3_} layer is an [Fe^II^(*μ*_3_-O)_3_(*μ*_4_-O)_3_] octahedron, which is surrounded by three {SbO_4_} structural units that connect to the central {FeO_6_} via edge-sharing a *μ*_3_-O(10) atom and a *μ*_4_-O(11). The average bond length of Fe-O and Sb-O in the layer is about 2.050 and 2.087 Å, respectively. Further, intermediate sandwich portions are connected by *μ*_3_-O atoms to the top and bottom layers {Sb^III^_3_(*μ*_3_-O)(tta)_3_}, respectively, and together they assemble into the anionic cluster of [Fe^II^_2_Sb^III^_12_(*μ*_3_-O)_8_(*μ*_4_-O)_3_(tta)_6_]^6−^ (Figure 2f). The counterions of [Co(H_2_O)_6_]^2+^ complexes, H_3_O^+^ and [Me_2_NH_2_]^+^ as well as lattice water molecules, wrap around the anionic cluster (Figure S1a,b). Compound **3** differs from compound **1** only in that the peripheral cation is changed from a [Co(H_2_O)_6_]^2+^ complex to a [Ni(H_2_O)_6_]^2+^ complex (Figure S1c,d).

**Figure 3:**
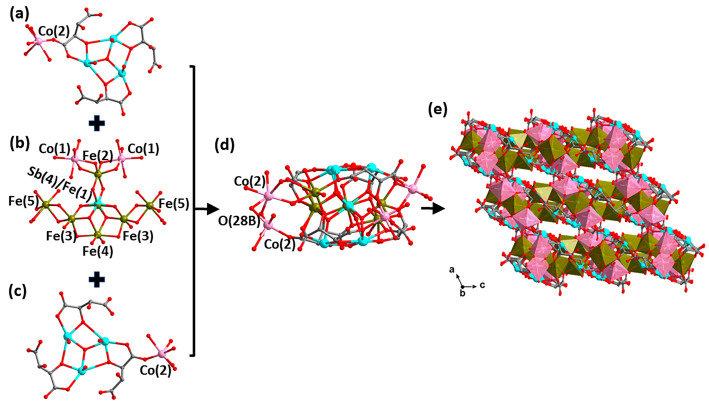
Structural evolution of the anion cluster [Co_2.5_Fe^II^_4/3_Fe^III^_3_(H_2_O)_13_Sb^V^_1/3_Fe^III^_2/3_(*μ*_4_-O)_2_(*μ*_3_-O)_4_Sb^III^_6_(*μ*_3_-O)_2_(tta)_6_]^5/3−^ in compound **2**. Structure of the top layer {Co_0.25_(H_2_O)_1.5_[Sb^III^_3_(*μ*_3_-O)(tta)_3_]} (**a**), the intermediate layer {Co_2_Fe^II^_4/3_Fe^III^_3_(H_2_O)_10_Sb^V^_1/3_Fe^III^_2/3_(*μ*_4_-O)_2_(*μ*_3_-O)_4_} (**b**), the bottom layer {Co_0.25_(H_2_O)_1.5_[Sb^III^_3_(*μ*_3_-O)(tta)_3_]} (**c**) and the anionic cluster {Co_2.5_Fe^II^_4/3_Fe^III^_3_(H_2_O)_13_Sb^V^_1/3_Fe^III^_2/3_(*μ*_4_-O)_2_(*μ*_3_-O)_4_Sb^III^_6_(*μ*_3_-O)_2_(tta)_6_}^5/3−^ (**d**). (**e**) The spatial stacking diagram showing the microporous structure of **2**. Color scheme: Fe, brown; Co, pink; Sb, turquoise; O, red and C, gray. Brown polyhedron: [FeO_x_] and pink polyhedron: [CoO_y_]. For clarity, hydrogen atoms are omitted.

Compound H_5/3_[Co_2.5_Fe^II^_4/3_Fe^III^_3_(H_2_O)_13_Sb^V^_1/3_Fe^III^_2/3_(*μ*_4_-O)_2_(*μ*_3_-O)_4_Sb^III^_6_(*μ*_3_-O)_2_(tta)_6_]·2H_2_O (**2**) crystallizes in the *C*2/*c* space group of monoclinic crystal system. The asymmetric unit of compound **2** includes half an anionic cluster of [Co_2.5_Fe^II^_4/3_Fe^III^_3_(H_2_O)_13_Sb^V^_1/3_Fe^III^_2/3_(*μ*_4_-O)_2_(*μ*_3_-O)_4_Sb^III^_6_(*μ*_3_-O)_2_(tta)_6_]^5/3−^, five-sixths of a hydrogen ion, and one lattice water molecule. Compound **2** is characterized by a monolayer sandwich type of a cluster with two symmetries. The top and bottom of the sandwich are both a {Co_0.25_(H_2_O)_1.5_[Sb^III^_3_(*μ*_3_-O)(tta)_3_]} layer (Figure 3a,c). The {Co_2_Fe^II^_4/3_Fe^III^_3_(H_2_O)_10_Sb^V^_1/3_Fe^III^_2/3_(*μ*_4_-O)_2_(*μ*_3_-O)_4_} layer occupies the intermediate sandwich (Figure 3b). In previously reported structures, the transition metals (e.g., Fe(II), Mn(II), Cu(II), etc.) occupy the center of the sandwich in majority, while occasionally the Sb(V) occupies the center [19,21]. By contrast, in compound **2** the center of the sandwich layer is occupied by the {Sb^V^_1/3_Fe^III^_2/3_(*μ*_4_-O)_2_(*μ*_3_-O)_4_} octahedron, with an average Sb-O and Fe-O bond length of about 2.004 and 2.002 Å, respectively. This is the first time that Sb(V) is co-occupied with the transition metal Fe(III) in transitional-metal-antimony-oxo tartrate cluster-based compounds. More interestingly, the successful preparation of compounds **2** and **4** makes the transformation process of the central metal of this type of monolayer sandwich from transition metal ions to Sb(V) ions clearer and more complete.

As shown in Figure 3b, two [Fe^III^(3)O_4_(*μ*_4_-O)(*μ*_3_-O)] octahedra and one [Fe^III^(2)O_4_(*μ*_3_-O)_2_] octahedron are connected to the central [Sb^V^_1/3_Fe^III^_2/3_(*μ*_4_-O)_2_(*μ*_3_-O)_4_] octahedron in an edge-sharing manner. The iron atoms of the interlayer (Fe(2) and Fe(3)) are hexa-coordinated with oxygen atoms, with an average bond length of about 2.007 (Fe(2)-O) and 2.032 (Fe(3)-O) Å, respectively. In addition, two [Co(1)O_6_] octahedra and two [Fe^II^(5)O_6_] polyhedra are connected to the [Fe(2)O_6_] and [Fe(3)O_6_] octahedra in a similar co-rimmed manner, respectively. Furthermore, there is a [Fe^II^(4)O_4_(*μ*_4_-O)_2_] octahedron connecting to two [Fe^III^(3)O_4_(*μ*_4_-O)(*μ*_3_-O)] octahedra as well as the [Sb^V^_1/3_Fe^III^_2/3_(*μ*_4_-O)_2_(*μ*_3_-O)_4_] octahedron together in a co-edged manner. All of the above polyhedra together form the central sandwich of {Co_2_Fe^II^_4/3_Fe^III^_3_(H_2_O)_10_Sb^V^_1/3_Fe^III^_2/3_(*μ*_4_-O)_2_(*μ*_3_-O)_4_}.

Then the central layer of {Co_2_Fe^II^_4/3_Fe^III^_3_(H_2_O)_10_Sb^V^_1/3_Fe^III^_2/3_(*μ*_4_-O)_2_(*μ*_3_-O)_4_} connects to the top and bottom {Co_0.25_(H_2_O)_1.5_[Sb^III^_3_(*μ*_3_-O)(tta)_3_]} layers via four *μ*_3_-O atoms and two *μ*_4_-O atoms to further assemble a sandwich structure (Figure 3d). Interestingly, the partially occupied [Co(2)_0.25_O_6_] as well as [Fe(5)_0.5_O_6_] polyhedra play the role of linkers, and thus the state of existence of the two groups affects the structural dimensions of the compounds. When none of the [Co(2)O_6_] octahedra are present, there is no way to further expand the anionic cluster portion, regardless of the presence or absence of [Fe(5)O_6_], at which point a discrete structure formed (Figure S2a); when only one of the [Co(2)O_6_] octahedra is present above or below, the discrete anionic cluster portion extends through [Co(2)O_6_] interconnections to form a 1-D chain structure (Figure S2b); when both [Co(2)O_6_] octahedra are present simultaneously, the 1-D chain is further interconnected through the [Co(2)O_6_] octahedra to form a 2-D layer structure (Figure S2c); however, when both [Co(2)O_6_] and [Fe(5)O_6_] polyhedra are present in the structure, the 2-D layer is further extended into a three-dimensional microporous structure by sharing O(28B) atoms through the [Co(2)O_6_] and [Fe(5)O_6_] polyhedra (Figure 3e). Regardless of the spatial stacking forms of compound **2**, hydrogen ions as well as lattice water molecules are always located around the anion clusters.

Compounds **2** and **4** are isostructural: the difference lies in that the cluster in compound **2** consists of Fe-Co-Sb while that in **4** is Fe-Ni-Sb, and the contents of the three elements Fe, Sb and M(Co/Ni) are slightly different (Figure S3). In compound **4**, the sandwich center is occupied by an [Sb^V^_0.25_Fe^III^_0.75_(*μ*_4_-O)_2_(*μ*_3_-O)_4_] octahedron; the content of Fe at the positions of Fe(4) and Fe(5) also differs slightly, with a slight increase in the Fe content at the position of Fe(5), which becomes [Fe^II^_0.715_(5)O_6_], and an even smaller amount of Fe at the position of Fe(4), which becomes [Fe^II^_0.07_(4)O_6_]; the [Ni(1)O_6_] octahedron and [Ni_0.125_(2)O_5_] replace the [Co(1)O_6_] as well as [Co_0.25_(2)O_6_] positions in compound **2**. The H^+^ ions surrounding the periphery of the cluster also change slightly from 5/3 per formula in **2** to 2 per formula in **4**. Although Ni(2) becomes five-coordination with O atoms in compound **4**, the way it is connected does change. However, the two [Ni_0.125_(5)O_5_] polyhedra hanging on the periphery are not connected to each other in an edge-sharing manner as that for the [Co_0.25_(2)O_6_] octahedra; instead, they hang separately in the top or bottom layer of the anionic cluster (Figure S4).

**Figure 4:**
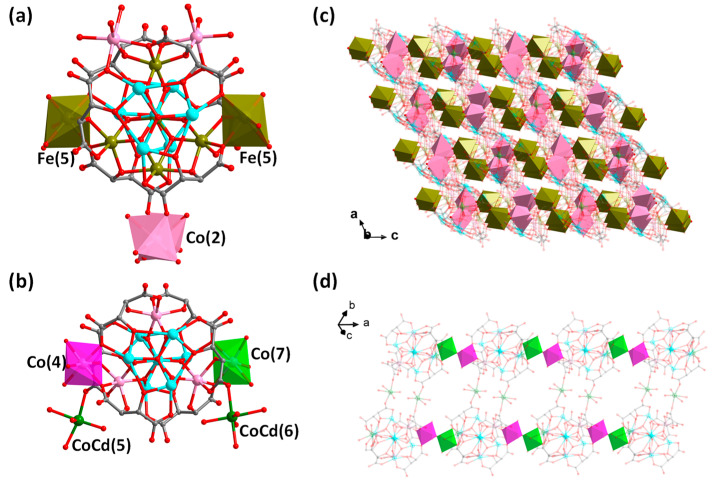
(**a**) Structure of the anionic cluster {Co_2. 5_Fe^II^_4/3_Fe^III^_3_(H_2_O)_13_Sb^V^_1/3_Fe^III^_2/3_(*μ*_4_-O)_2_(*μ*_3_-O)_4_Sb^III^_6_(*μ*_3_-O)_2_(tta)_6_}^5/3−^ in compound **2**. (**b**) Structure of the anionic cluster of {*M*_0.5_(H_2_O)_3.5_{*M*’(H_2_O)_4_[Sb^V^O_6_[Co_4.2_(H_2_O)_3_Sb^III^_6_(*μ*_3_-O)_2_(tta)_6_]}}^5.6−^ ({Cd[SbCo]}) [21]. (**c**) Packing of the anionic 3-D framework of **2** viewed along the *b*-axis. Brown polyhedron: [Fe(5)O_6_]; pink polyhedron: [Co(2)O_6_]. (**d**) 1-D belt-like structure of {Cd[SbCo]}*_n_*. Purple polyhedron: [Co(4)O_6_] and green polyhedron: [Co(7)O_6_]. Color scheme: Fe, brown; Co, pink; Sb, turquoise; CoCd, green; O, red and C, gray. For clarity, hydrogen atoms are omitted and some atoms as well as bonds are 80% transparent.

Figure 4 further compares the structure of compound **2** with that of compound {*M*_0.5_(H_2_O)_3.5_{*M*’(H_2_O)_4_[Sb^V^O_6_[Co_4.2_(H_2_O)_3_Sb^III^_6_(*μ*_3_-O)_2_(tta)_6_]}}^5.6−^ (*M* = Cd_0.39_/Co_0.61_, *M*’ = Cd_0.24_/Co_0.76_, abbreviated as {Cd[SbCo]}) we just reported [21]. As can be seen in Figure 4a, the metal in the intermediate sandwich portion has been changed from the original {Sb^V^O_6_Co_3_Co(4)Co_0.2_(7)} in {Cd[SbCo]} to {Sb^V^_1/3_Fe^III^_2/3_O_6_Fe_3_Co_2_(Fe_0.5_(5))_2_Fe^II^_1/3_(4)} in **2**. In compound **2**, [Co(2)O_6_] is statistically distributed on the top or bottom {Sb^III^_3_(*μ*_3_-O)(tta)_3_} layer of the anion cluster and connects neighboring anion cluster units, whereas in compound {Cd[SbCo]}, CoCd(5) and CoCd(6) it hangs in the top or bottom {Sb^III^_3_(*μ*_3_-O)(tta)_3_} layers, respectively, and only CoCd(5) connects the neighboring anionic cluster to form the dimeric units, and CoCd(6) does not serve as connecting units (Figure 4b). In addition, the two anionic clusters are connected and extended in different ways; compound **2** extends the cluster into a 3-D microporous structure by sharing O(28B) through partially occupied Fe(5) with Co(2) suspended at the periphery of the cluster (Figure 4c); whereas, the anionic cluster unit of {Cd[SbCo]} connects to each other through partially occupied Co(7) and Co(4) to form an 1-D chain, which in turn forms an 1-D belt-like structure through the bridging of two CoCd(5) compounds (Figure 4d). Therefore, it is expected that one can intentionally regulate the composition of the intermediate layer and connecting units at the periphery of the sandwich-typed antimony-oxo tartrate cluster to further construct more novel structures in terms of cluster size/composition, structural dimensionality and functionality, etc.

### 2.3. Thermal Stability and UV-Vis Analysis

Prior to testing, all compounds were subjected to powder diffraction characterization and compared to simulated diffraction peaks obtained from single-crystal diffraction data to demonstrate the purity of the compounds (Figure S5). We ground the crystals of compounds **2** or **4** into powder and immersed them in water for 1 day, the powder diffraction results showed that both compounds were stable in water for 1 day (Figure S5b,d). The thermal stability of all compounds was also investigated (Figure S6). As shown in Figure S6, compounds **1** and **3** lost eighteen water molecules (six lattice and twelve coordination water molecules) and one H_3_O^+^ as well as one dimethylammonium cation in the range of room temperature to 275 °C, respectively (calcd. 12.43%, found 12.02% (**1**) and calcd. 12.44%, found 12.59% (**3**)); compounds **2** and **4** lost fifteen (thirteen lattice and two coordination water molecules) and sixteen (fourteen lattice and two coordination water molecules) water molecules, respectively (calcd. 10.92%, found 11.22% (**2**) and calcd. 11.60%, found 11.62% (**4**)), in the temperature range of RT to 250 °C. In addition, we performed solid-state-UV-diffuse-reflectance measurements for all compounds, and the band gaps of 2.69 eV (**1**), 2.29 eV (**2**), 2.58 eV (**3**) and 2.32 eV (**4**) were ascertained from the fitting results of compounds **1**-**4**, respectively, which are consistent with the colors of the corresponding compounds (Figure S7).

### 2.4. X-ray Photoelectron Spectroscopy (XPS) Analysis of ***2*** and ***4***

**Figure 5:**
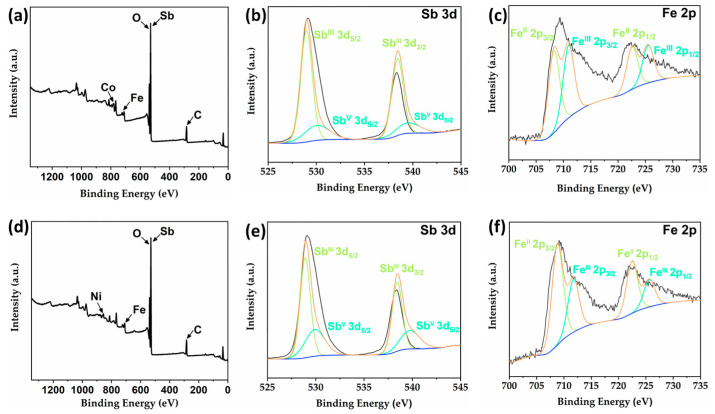
(**a**) The survey XPS spectrum of compound **2**. High-resolution XPS spectra of Sb 3d (**b**) and Fe 2p (**c**) for compound **2**. (**d**) The survey XPS spectrum of compound **4**. High-resolution XPS spectra of Sb 3d (**e**) and Fe 2p (**f**) for compound **4**.

When resolving the structures of compounds **2** and **4**, we determined the valence of Fe as well as Sb in the structures based on reported compounds with the same type. To further verify the accuracy of our results, we analyzed these two compounds via X-ray photoelectron spectroscopy (XPS) and bond valence calculations. The valence states calculation results show that all the Sb atoms in compounds **2** and **4** have a valence state of +3 except for the Sb(4) atom which has a valence state of +5; the valence states of Fe(1), Fe(2) and Fe(3) atoms are +3, while the valence states of Fe(4) and Fe(5) atoms are +2 (Table S2) [13,16,17,22]. XPS results of compounds **2** and **4** further support our conclusions (Figure 5). In the survey spectra of compounds **2** and **4** (Figure 5a,d), the characteristic peaks of Fe, Co, Sb, C, O and Fe, Ni, Sb, C, O elements have been detected, respectively, which are consistent with the compositions of these two compounds. As shown in Figure 5b,e, the high-resolution XPS spectra of Sb 3d can all be fitted to two pairs of peaks; the split peaks at 528.98 and 538.43 eV (**2**) as well as 528.92 and 538.43 eV (**4**) correspond to Sb^III^ 3d_5/2_ and Sb^III^ 3d_3/2_, respectively, while the peaks at 530.06 and 539.51 eV (**2**) and 529.92 and 539.76 eV correspond to Sb^V^ 3d_5/2_ and Sb^V^ 3d_3/2_, respectively. Similarly, the high-resolution XPS pattern of Fe 2p can be fitted to two pairs of peaks, and the characteristic peaks located at 708.26 and 722.57 (**2**) eV as well as 708.81 and 722.47 eV (**4**) correspond to Fe^II^ 2p_3/2_ and Fe^II^ 2p_1/2_, respectively, while the split peaks located at 710.96 and 725.51 eV (**2**) and 711.75 and 725.62 eV (**4**) correspond to Fe^III^ 2p_3/2_ and Fe^III^ 2p_1/2_, respectively (Figure 5c,f). The above XPS results are in agreement with those reported in the literature, which further confirms the coexistence of +3 and +5 valence Sb atoms and +2 and +3 valence Fe atoms in **2** and **4** [23,24]. Furthermore, to the best of our knowledge, this is one of the few examples of heterometallic antimony-oxo clusters containing mixed valence Sb(III, V) [19,21].

### 2.5. Photodegradation Performance of Compounds ***2*** and ***4***

**Figure 6:**
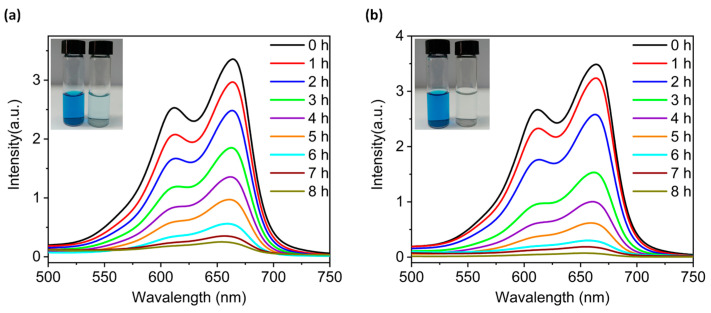
Liquid UV-visible absorption spectra of methylene blue (MB) solution containing compounds **2** (**a**) and **4** (**b**) over the varying illumination time. The initial concentration of MB solution was 30 ppm and the test temperature was 25 °C. The insets in (**a**,**b**) are the photos of MB solution photodegraded with compounds **2** and **4** for 8 h.

The vigorous development of printing, leather and other industries has generated a large amount of wastewater containing organic dye pollutants. Thus, it is crucial to solve the environmental pollution problems caused by these species [25,26,27]. In recent years, a number of scholars have utilized metal oxides with precise atomic structures as catalysts for photodegradation of organic dye wastewater [8,28,29]. On account of this, we investigated the photocatalytic degradation performance of compounds **2** and **4** as inhomogeneous reaction photocatalysts using methylene blue (MB) as a simulated pollutant. The photodegradation performance of MB solution after different times of light exposure in absence of a photocatalyst was measured with a UV-Vis spectrophotometer. According to the regression equations of the standard solution curves (Figure S8a), the degradation rate of the MB solution was only 23.07% (Figure S8b). Under the dark condition, the degradation rate of MB was less than 10% even in the presence of compounds **2** or **4** (Figure S8c–f). However, in the presence of compounds **2** and **4**, the intensity of the characteristic absorption peak at 667 nm of the MB solution continuedly decreased with prolongation of light time (Figure 6). The degradation rates of MB solution via compounds **2** and **4** after 8 h of light exposure are calculated to be 93.58% (**2**) and 98.30% (**4**), respectively. The above results together prove that compounds **2** and **4** can act as the visible-light-driven photocatalysts to photocatalyze the degradation of MB solution. Furthermore, X-ray powder diffraction results also prove that the structures of **2** and **4** remained stable during the degradations (Figure S5b,d).

### 2.6. Proton Conduction of Compounds ***2*** and ***4***

**Figure 7:**
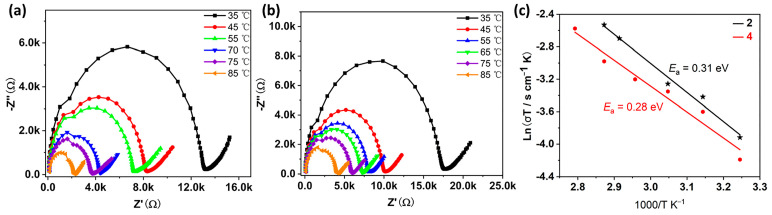
Nyquist plots from AC impedance data of **2** (**a**) and **4** (**b**) at 98% RH and varied temperatures between 35 and 85 °C. (**c**) Arrhenius plots of proton conductivity of compounds **2** and **4** at different temperatures under 98% RH.

In previous work, we have demonstrated that such antimony tartrate cluster-based compounds have the potential to act as proton conducting materials [15,19,21]. Given that compounds **2** and **4** can be stabilized in water, we evaluated the proton conductivity of columnar powder samples of the two compounds at different temperatures with a relative humidity of 98%. As shown in Figure 7, the resistance of the two examples of compounds decreases with increasing temperature, indirectly indicating that the proton conductivity of the compounds also increases. The proton conductivity was measured to be 1.81 × 10^−5^ S·cm^−1^ for **2** and 1.39 × 10^−5^ S·cm^−1^ for **4**, respectively, when the temperature was 25 °C and the relative humidity was 98% (Figures S9 and S10). The proton conductivity continued to increase as the temperature raised and reached a maximum of 3.86 × 10^−4^ S·cm^−1^ for **2** and 2.12 × 10^−4^ S·cm^−1^ for **4** (Table S3), respectively, when the temperature reached 85 °C at 98% RH. The increase in temperature makes the water molecules move at a faster rate which may be responsible for the increase in proton conductivity. Compared to our previously reported antimony tartrate-based clusters of the same type, these two compounds show slightly lower proton conductivities, which may be due to the different contents of lattice water molecules.

Based on the proton conductivity at different temperature, we calculated the activation energies regarding the proton conduction process of the two compounds, which are 0.31 eV and 0.28 eV, respectively (Figure 7c). According to previous studies, when the activation energy is less than 0.5 eV, the conduction mechanism is a Grothuss mechanism [30,31,32]. In addition, the powder diffraction results after the proton conduction test show that the structures of the compounds remained stable (Figure S5b,d).

### 2.7. Magnetic Measurements of Compounds ***2*** and ***4***

In the reported antimony tartrate-based clusters, the intercalation metals are basically first transition metal ions (e.g., V, Cr, Mn, Fe, Co, Ni and Cu), and their magnetic properties have been studied [13,14,16,17,18,20]. For example, the magnetic properties of the compound H_5_K_3_[(CH_3_)_2_NH_2_]_2_[Fe_7_Sb_6_(*μ*_4_-O)_6_(*μ*_3_-O)_2_(L-tta)_6_]_2_·28H_2_O were determined in the range of 5–300 K, suggesting that the compound has predominantly an antiferromagnetic exchange interaction [13]; the magnetic properties of the compound Na_6_[Cu_7_Sb_6_(*μ*_3_-OH)_2_(*μ*_4_-O)_6_(L-tta)_6_]·24H_2_O were measured, indicating that the compound is ferromagnetic [14]. In view of this, we have investigated the magnetic behaviors of **2** and **4**.

**Figure 8:**
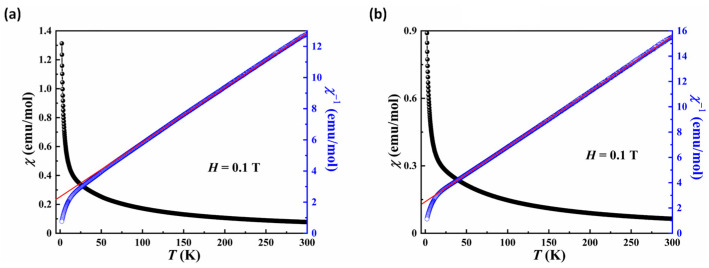
Plots of the χ and χ^−1^ versus T for compounds **2** (**a**) and **4** (**b**) in the 2~300 K temperature range in an applied field of H = 0.1 T. The red line indicates Curie-Weiss fitting.

Figure 8 illustrates temperature dependence of magnetic susceptibility and the corresponding reciprocal one obtained at *H* = 0.1 T for **2** and **4**. Both compounds exhibited analogous magnetic behavior. Their susceptibility increased with decreasing temperature and no peaks were detected down to 2 K, indicating a paramagnetic behavior at low temperature. It is noted that the susceptibility between 2 and 300 K could be well-fitted with the Curie–Weiss law *χ* = *χ*_0_ + *C*/(*T* − *θ*_CW_) above 100 K, giving *χ*_0_ = 0.0049(8) and −0.0013(1), the Curie constant *C* = 25.75(3) and 23.43(4) cm^3^·K·mol^−1^, Weiss constant *θ*_CW_ = −54.14(6) and −58.58(3) K for compounds **2** and **4**, respectively. The effective magnetic moment of **2** and **4** was calculated as 14.35 *μ*_B_ and 13.69 *μ*_B_ according to *μ*_eff_^2^ = 8*C*, respectively, which is close to the theoretical values of 14.07 *μ*_B_ (11/3Fe^3+^, 4/3Fe^2+^ and 2.5Co^2+^) and 13.19 *μ*_B_ (3.75Fe^3+^, 1.5Fe^2+^ and 2.25Ni^2+^), respectively. Furthermore, the negative Weiss temperature indicates the dominative antiferromagnetic interactions between neighboring magnetic ions for **2** and **4**.

## 3. Materials and Methods

All reagents for synthesis were purchased from commercial sources and used without further purification.

**Synthesis of compounds 1 and 2:** A mixture of K_2_Sb_2_(tta)_2_·3H_2_O (100.0 mg and 0.15 mmol), Co(NO_3_)_2_·6H_2_O (87.3 mg and 0.3 mmol), FeSO_4_·7H_2_O (55.6 mg and 0.2 mmol), 3 mL H_2_O and 1 mL DMF was sealed in a 8 mL vial, and then transferred to a preheated oven at 100 °C for 4 days. After cooling to room temperature, the product was washed several times with water and anhydrous ethanol. After filtering and drying at room temperature, 9.3 mg dark-green hexagonal prismatic crystals (**1**) (Yield: 5.05%, based on Fe) as well as 58.4 mg brownish-yellow flaky crystals (**2**) (Yield: 58.91%, based on Fe) were obtained by hand picking. EA (Elemental analysis), anal. calcd. for C_26_H_59_O_66_NFe_2_Co_2_Sb_12_ of compound **1**: C, 9.97; H, 1.90; N, 0.45%; found: C, 10.15; H, 1.98; N, 0.47%. calcd. for C_24_H_47.33_O_59_Fe_5_Co_2.5_Sb_6.33_ of compound **2**: C, 11.65; H, 1.78%; found: C, 11.51; H, 1.98%. ICP, anal. calcd. for C_24_H_47.33_O_59_Fe_5_Co_2.5_Sb_6.33_ of compound **2**: Fe, 11.31; Co, 5.97; Sb, 31.06%; found: Fe, 9.86; Co, 7.23; Sb, 27.64%.

Thiophene-2,5-dicarboxylic acid (50.0 mg and 0.29 mmol) or pyrazole-3,5-dicarboxylic acid (50.0 mg and 0.29 mmol) was added to the above synthesis while keeping other conditions unchanged, and the product was washed several times with water and anhydrous ethanol. Finally, 15.3 mg of compound **1** (Yield: 8.31%, based on Fe) was obtained.

**Synthesis of compounds 3 and 4:** The synthesis of **3** and **4** is similar to that of **1** and **2**, except for replacing Ni(NO_3_)_2_·6H_2_O (87.2 mg and 0.3 mmol) with Co(NO_3_)_2_·6H_2_O (87.3 mg and 0.3 mmol), and 14.9 mg dark-green hexagonal prismatic massive crystals (**3**) (Yield: 8.08%, based on Fe) as well as 60.3 mg brownish-yellowish-green flaky crystals (**4**) (Yield: 63.74%, based on Fe) were obtained. EA, anal. calcd for C_26_H_59_O_66_NFe_2_Ni_2_Sb_12_ of compound **3**: C, 9.97; H, 1.90; N, 0.45%; found: C, 10.12; H, 2.00; N, 0.46%. calcd. for C_24_H_49.75_O_60_Fe_5.25_Ni_2.25_Sb_6.25_ of compound **4**: C, 11.62; H, 1.87%; found: C, 11.59; H, 1.99%. ICP, anal. calcd. for C_24_H_49.75_O_60_Fe_5.25_Ni_2.25_Sb_6.25_ of compound **4**: Fe, 11.81; Ni, 5.32; Sb, 30.68%; found: Fe, 11.14; Ni, 5.92; Sb, 29.26%.

Similarly, 16.7 mg of compound **2** was obtained by adding thiophene-2,5-dicarboxylic acid (50.0 mg and 0.29 mmol) or pyrazole-3,5-dicarboxylic acid (50.0 mg and 0.29 mmol) to the above synthesis (Yield: 9.06%, based on Fe).

**Single-Crystal X-ray diffraction (SCXRD):** Crystals of appropriate size and dimensions were selected under a microscope and then the crystals were fixed at the tip with a glass wire for single-crystal X-ray diffraction (SCXRD) characterization. SCXRD data for all the four title compounds were collected on a SuperNova CCD diffractometer with graphite monochromatic Mo*K*_α_ radiation (λ = 0.71073 Å). The collection temperature of the title compounds is 297 (2) K. Crystallographic data and refinement details for **1**, **2**, **3** and **4** are shown in Table S4. Selected bond lengths and angles of compounds **1**, **2**, **3** and **4** are shown in Table S5. EA, TGA, EDS, ICP and XPS together verified the empirical formulas (Figure 5, Figure S6 and Figure S11).

CCDC Nos. 2313706 for **1**, 2313704 for **2**, 2313703 for **3** and 2313708 for **4** contain the supplementary crystallographic data. The structures were solved via direct methods and refined with full-matrix least-squares on *F*^2^ using the SHELX-2018 program package [33].

## 4. Conclusions

In summary, we have synthesized four examples of transition-metal-antimony-oxo tartrate cluster-based compounds (H_3_O)(Me_2_NH_2_)[M(H_2_O)_6_]_2_[Fe_2_Sb_12_O_11_(tta)_6_]·6H_2_O (M = Co (**1**); M = Ni (**3**)), H_5/3_[Co_2.5_Fe^II^_4/3_Fe^III^_3_(H_2_O)_13_Sb^V^_1/3_Fe^III^_2/3_(*μ*_4_-O)_2_(*μ*_3_-O)_4_Sb^III^_6_(*μ*_3_-O)_2_(tta)_6_]·2H_2_O (**2**) and H_2_[Ni_2.25_Fe^II^_1.5_Fe^III^_3_(H_2_O)_14_Sb^V^_0.25_Fe^III^_0.75_(*μ*_4_-O)_2_(*μ*_3_-O)_4_Sb^III^_6_(*μ*_3_-O)_2_(tta)_6_]·2H_2_O (**4**) via a simple solvothermal method. All four compounds belong to the sandwich conformation; the top and bottom layers of them are all {Sb^III^_3_(*μ*_3_-O)(tta)_3_} scaffolds, while the intermediate layer of **1** and **3** is a {Fe(*μ*_3_-O)_3_(*μ*_4_-O)_3_Sb_3_}_2_ bilayer, and that for **2** and **4** is a monolayer of {Co_2_Fe^II^_4/3_Fe^III^_3_(H_2_O)_10_Sb^V^_1/3_Fe^III^_2/3_(*μ*_4_-O)_2_(*μ*_3_-O)_4_} and {Ni_2_Fe^II^_1.5_Fe^III^_3_(H_2_O)_11_Sb^V^_0.25_Fe^III^_0.75_(*μ*_4_-O)_2_(*μ*_3_-O)_4_}, respectively. It is interesting to note that the center portions of the intermediate layer of **2** and **4** are occupied by [Sb^V^_1/3_Fe^III^_2/3_(*μ*_4_-O)_2_(*μ*_3_-O)_4_] and [Sb^V^_0.25_Fe^III^_0.75_(*μ*_4_-O)_2_(*μ*_3_-O)_4_] octahedrons, respectively, demonstrating that these two compounds appear to have a rare mixed valence state of Sb(III,V). In addition, we have captured the intermediate [Fe^III^_x_Sb^V^_1−x_O_6_] of the center metal from the simple transition metal [Fe^III^O_6_] to [Sb^V^O_6_], and thus completed the process of this change. We have also carried out studies related to photocatalytic degradation of MB solutions, proton conduction and magnetic properties for compounds **2** and **4**, and the experimental results have shown that they are a class of multifunctional materials. In the future, we will adjust the experimental conditions to combine compounds **2** or **4** with organic carboxylic acid ligands to construct 2-D layered or 3-D framework structures with pores and then explore their potential in proton conductivity and ion exchange.

## Data Availability

Data are contained within the article and Supplementary Materials.

## Supplementary Materials

The following supporting information can be downloaded at: https://www.mdpi.com/article/10.3390/molecules29030591/s1. Table S1. Reported studies on the structure and properties of transition-metal-antimony-oxo tartrate cluster-based compounds; Figure S1. Structure of compounds **1** (a) and **3** (c) and packing diagrams of compounds **1** (b) and **3** (d). For clarity, hydrogen atoms, H_3_O^+^ and [Me_2_NH_2_]^+^ cations are omitted. Color scheme: Fe, brown; Sb, turquoise; Co, pink; Ni, green; O, red and C, gray; Figure S2. Packing of 0-D isolated clusters in **2**. The 1-D chain (b) and 2-D layer (c) in **2**. For clarity, hydrogen atoms are omitted. Color scheme: Fe, brown; Sb, turquoise; Co, pink; O, red and C, gray; Figure S3. Structure of compound **4**. For clarity, hydrogen atoms are omitted. Color scheme: Fe, brown; Sb, turquoise; Ni, green; O, red and C, gray; Figure S4. Packing diagram of compound **4**. For clarity, hydrogen atoms and H_3_O^+^ cations are omitted; Figure S5. Simulated and observed PXRD patterns of compounds **1** (a), **2** (b), **3** (c) and **4** (d); Figure S6. Thermogravimetric curves for compounds **1** (a), **2** (b), **3** (c) and **4** (d); Figure S7. Solid-state UV-Vis absorption spectra of compounds **1** (a), **2** (b), **3** (c) and **4** (d); Table S2. The bond valence sum calculations for compounds **2** and **4**. Figure S8. (a) Linearity of absorbance (*A*) versus standard concentration (*C*) of MB solution. (b) Curve of *C_t_*/C_0_ of MB solution with time (*t*) in the absence of photocatalyst. Liquid UV-visible absorption spectra of MB solution containing compound **2** (c) and compound **4** (d) under dark conditions at different times. *C_t_*/C_0_ curves of MB solution for compounds **2** (e) and **4** (f) as a function of time (*t*) under dark (black line) or light (blue line) conditions; Figure S9. The Nyquist plot from AC impedance data of **2** at 98% RH and 25 °C; Figure S10. The Nyquist plot from AC impedance data of **4** at 98% RH and 25 °C; Table S3. Proton conductivity (*σ*) values of **2** and **4** under 98% RH at different temperatures; Figure S11. EDS results of compounds **1** (a), **2** (b), **3** (c) and **4** (d); Table S4. Crystallographic data and refinement details for **1**, **2**, **3** and **4**; Table S5. Selected bond lengths (Å) and angles (°) of compounds **1**, **2**, **3** and **4**.
