# Supplementary material for: Synthesis and Properties of Cobalt/Nickel-Iron-Antimony(III, V)-Oxo Tartrate Cluster-Based Compounds
Source: Molecules. 2024 Jan 25;29(3):591. doi: 10.3390/molecules29030591 (PMC10856786; doi:10.3390/molecules29030591)
Supplement: Supplementary file 1 [file molecules-29-00591-s001.zip › molecules-2795923-supplementary.pdf]

Synthesis and properties of cobalt/nickel-iron-antimony(III, V)-oxo tartrate cluster-based compounds

Wei-Yang Wen [1,2], Yan-Qi Wang [5], Tian-Yu Pan [1,2], Qian-Qian Hu [1,3], Hui-Ping Xiao [2], Nan-Nan Wang [4], Xiao-Qi Li [4], Xin-Xiong Li [2], Bing Hu [1,3]* and Xiao-Ying Huang [1,3]*

1 State Key Laboratory of Structural Chemistry, Fujian Institute of Research on the Structure of Matter, Chinese Academy of Sciences, Fuzhou 350002, China

2 College of Chemistry, Fuzhou University, Fuzhou 350108, China

3 University of Chinese Academy of Sciences, Beijing 100049, China

4 Fujian Provincial Key Laboratory of Advanced Materials Oriented Chemical Engineering, College of Chemistry and Materials Science, Fujian Normal University, Fuzhou 350007, China

5 Fujian Provincial Key Laboratory of Ecology-Toxicological Effects & Control for Emerging Contaminants, College of Environmental and Biological Engineering, Putian University, Putian 351100, China

* Correspondence: hubing@fjirsm.ac.cn (B.H.); xyhuang@fjirsm.ac.cn (X.-Y.H.); Tel.: +86-591-6317-3145 (X.-Y.H.)

Table S1. Reported studies on the structure and properties of transition-metal-antimony-oxo tartrate cluster-based compounds.

structural formula	configuration	intermediate layer	property	ref
$H_3Na_7[Fe_4Sb_6(\mu_4\text{-}O)_6(\mu_3\text{-}O)_2(L\text{-}tta)_6]\cdot 14H_2O$	monolayer	$\{FeO_6Fe_3\}$	magnetism	13
$H_5K_3[(CH_3)_2NH_2]_2[Fe_7Sb_6(\mu_4\text{-}O)_6(\mu_3\text{-}O)_2(L\text{-}tta)_6]_2\cdot 28H_2O$	monolayer	$\{FeO_6Fe_3Fe_3\}$	magnetism	13
$Na_3K_5[(CH_3)_2NH_2]_4[Fe_6Sb_6(\mu_4\text{-}O)_6(\mu_3\text{-}O)_2(L\text{-}tta)_6]_2\cdot 30H_2O$	monolayer	$\{FeO_6Fe_3Fe_2\}$		13
$(H_4TPOM)[Fe_4Mn_3Sb_6(\mu_4\text{-}O)_6(\mu_3\text{-}O)_2(L\text{-}tta)_6(H_2O)]\cdot 16H_2O$	monolayer	$\{FeO_6Fe_3Mn_3\}$		17
$Mn(H_2O)_6[Fe_4Mn_4Sb_6(\mu_4\text{-}O)_6(\mu_3\text{-}O)_2(L\text{-}tta)_6(H2O)_8]\cdot 10.5H_2O$	monolayer	$\{FeO_6Fe_3Mn_4\}$	magnetism	16
$[V_4Mn_5Sb_6(\mu_4\text{-}O)_6(\mu_3\text{-}O)_2(L\text{-}tta)_6(H_2O)_{13}]\cdot 9.5H_2O$	monolayer	$\{VO_6V_3Mn_5\}$	magnetism	16
$Na_6[Cu_7Sb_6(\mu_3\text{–}OH)_2(\mu_4\text{-}O)_6(L\text{-}tta)_6]\cdot 24H_2O$	monolayer	$\{CuO_6Cu_3Cu_3\}$	magnetism	14
$Na_6[MnCu_6Sb_6(\mu_3\text{-}OH)_2(OH)(\mu_4\text{-}O)_6(tta)_6]\cdot 20H_2O$	monolayer	$\{MnO_6Cu_3Cu_3\}$	magnetism	20
$H_3K_5(H_2O)_{11}\{Cd(H_2O)_4[Cd(H_2O)Fe_4Cd_2Sb_6(\mu_4\text{-}O)_5(\mu_3\text{-}O)_3$ $(L\text{-}tta)_6][Cd(H_2O)_2Fe_4Cd_2Sb_6(\mu_4\text{-}O)_4(\mu_3\text{-}O)_4(L\text{-}tta)_6]$ $[Cd(H_2O)_2Fe_4Cd_2Sb_6(\mu_4\text{-}O)_4(\mu_3\text{-}O)_4(L\text{-}tta)_6Cd(H_2O)_5]\}\cdot 17H_2O$	monolayer	$\{FeO_6Fe_3Cd_3\}$	proton conduction	19
$H_5\{ACd(H_2O)_6[A(H_2O)_3Co_3Sb^{V}Sb^{III}_6(\mu_3\text{-}O)_8(L\text{-}tta)_6]\}\cdot 7H_2O$ $(A = Cd_{0.5} + Co_{0.5})$	monolayer	$\{Sb^{V}O_6Co_3Co_{0.5}Cd_{0.5}\}$	proton conduction	19
$H_{9.2}[Co(H_2O)_6]\{Co_{0.5}(H_2O)_{3.5}\{Co(H_2O)_4[Sb^{V}O_6[Co_{4.2}(H_2O)_5$ $Sb^{III}{}_6(\mu_3\text{-}O)_2(tta)_6]]\}\}_2\cdot 9H_2O$	monolayer			21
$H_{9.2}[Co(H_2O)_6]\{M_{0.5}(H_2O)_{3.5}\{M'(H_2O)_4[Sb^{V}O_6[Co_{4.2}(H_2O)_5$ $Sb^{III}{}_6(\mu_3\text{-}O)_2(tta)_6]]\}\}_2\cdot 7H_2O$ $(M = Cd_{0.39}+Co_{0.61},\ M' = Cd_{0.24}+Co_{0.76})$	monolayer	$\{Sb^{V}O_6Co_3Co_{1.2}\}$	proton conduction	21
$(H_3O)[Ni(H_2O)_6]_2[NiCrSb_{12}(\mu_3\text{-}O)_8(\mu_4\text{-}O)_3(L\text{-}tta)_6]\cdot 6H_2O$	bilayer	$\{NiCrO_9Sb_6\}$	magnetism	16
$Na_4[Cu_2Sb12(\mu_3\text{-}O)_6(\mu_3\text{-}OH)_2(\mu_4\text{-}O)_3(L\text{-}tta)_6]\cdot 19H_2O$	bilayer	$\{Cu_2O_9Sb_6\}$	magnetism	14
$\{Na_4[Co_2Sb_{12}(\mu_3\text{-}O)_6(\mu_3\text{-}OH)_2(\mu_4\text{-}\ O)_3(tta)_6]\cdot 19.5H_2O\}_2$	bilayer	$\{Co_2O_9Sb_6\}$	magnetism	18
$\{KNa_3[Ni_2Sb_{12}(\mu_3\text{-}O)_5(\mu_3\text{-}OH)_3(\mu_4\text{-}O)_3(tta)_6]\cdot 20H_2O\}_2$	bilayer	$\{Ni_2O_9Sb_6\}$	magnetism	18
$(H_3O)_7[Cd_7Sb_{24}O_{24}(L\text{-}tta)_9(L\text{-}Htta)_3(H_2O)_6]\cdot 29H_2O$	bowl-shaped		proton conduction, ion exchange	15

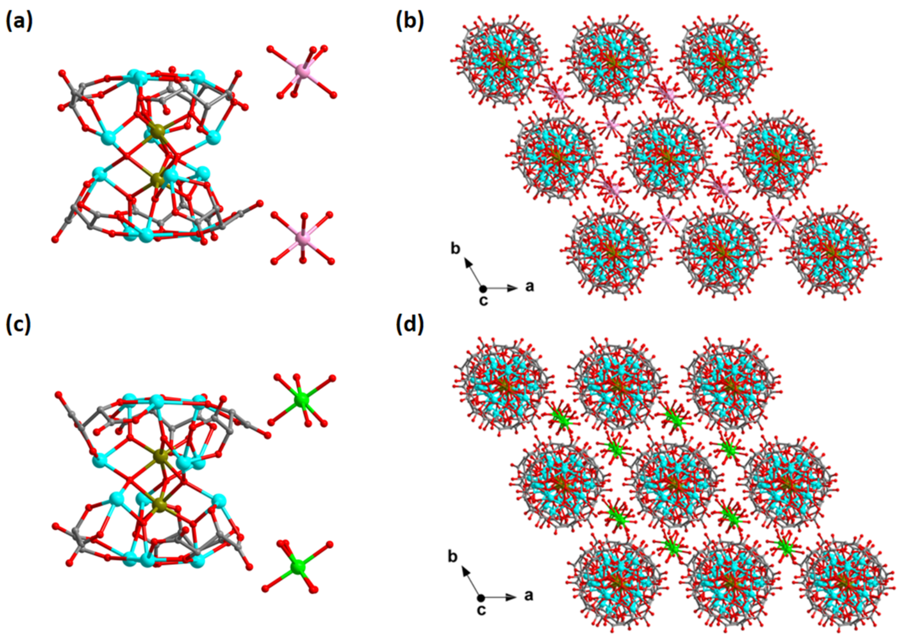

Figure S1. Structure of compounds **1** (a) and **3** (c); packing diagrams of compounds **1** (b) and **3** (d). For clarity, hydrogen atoms, H_3O^+ and $[Me_2NH_2]^+$ cations are omitted. Color scheme: Fe, brown; Sb, turquoise; Co, pink; Ni, green; O, red; C, gray.

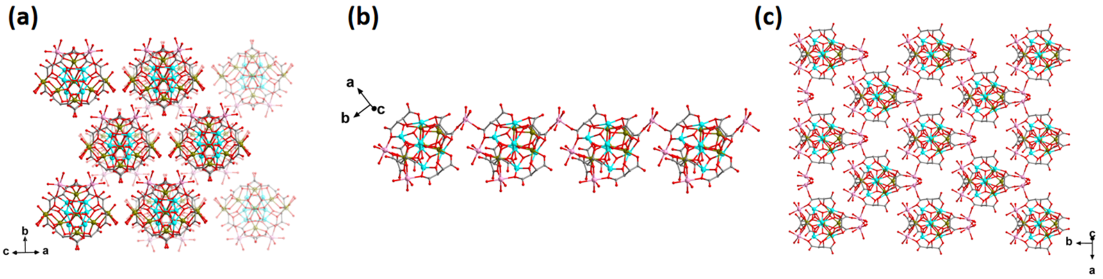

Figure S2. (a) Packing of 0-D isolated clusters in **2**. The 1-D chain (b) and 2-D layer (c) in **2**. For clarity, hydrogen atoms are omitted. Color scheme: Fe, brown; Sb, turquoise; Co, pink; O, red; C, gray.

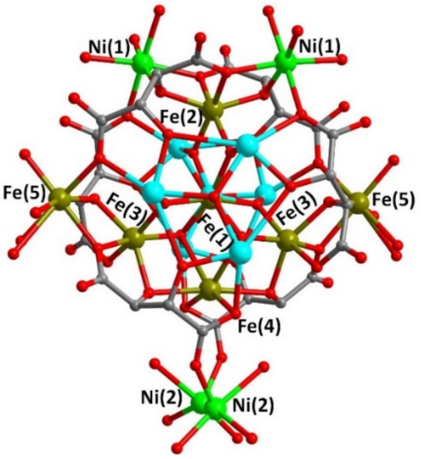

Figure S3. Structure of compound **4**. For clarity, hydrogen atoms are omitted. Color scheme: Fe, brown; Sb, turquoise; Ni, green; O, red; C, gray.

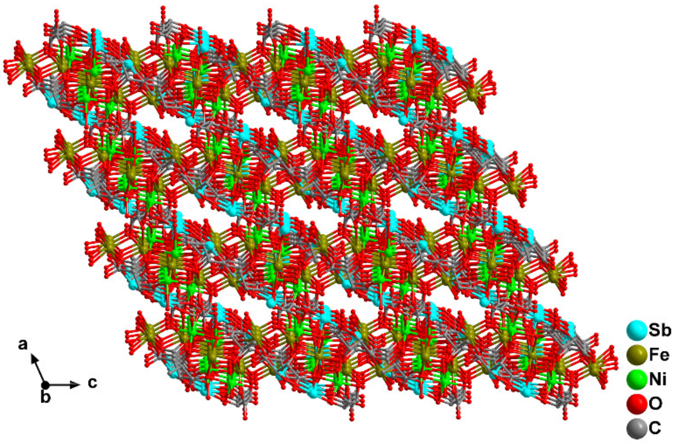

Figure S4. Packing diagram of compound **4**. For clarity, hydrogen atoms and H_3O^+ cations are omitted.

Physical measurements

Powder X-ray diffraction (PXRD) patterns were obtained on a Rigaku Miniflex-II diffractometer by using CuK_α radiation (λ = 1.54178 Å). Measurements were carried out in the 2-theta range of 3–65° by employing a step size of 0.02° with an integration time of 1 s. The UV-vis absorption spectra of the solid samples were recorded on a Shimadzu 2600 UV/vis spectrometer in the range of 200−800 nm at room temperature. A $BaSO_4$ plate was utilized as a standard which possesses 100% reflectance. The absorption data were then obtained from the reflectance spectra by using the Kubelka-Munk function $\alpha/S= (1-R)^2/2R$, where α refers to the absorption coefficient, S refers to the scattering coefficient, and R refers to the reflectance. Thermogravimetric analyses were performed using crystalline samples loads in a Al_2O_3 crucible with a NETZSCH STA 449F3 unit at a heating rate of 10 K·min^{-1} under N_2 atmosphere and in the temperature range of 20–800 °C. Energy dispersive spectrometry (EDS) analyses were performed using a JEOL JSM-6700F type desktop scanning electron microscope and a HITACHI FE-SEM SU8010 scanning electron microscope. Elemental analyses of C, H, and N were carried out with an Element Vario EL III elemental analyzer. The metal elemental ratios were determined by inductively coupled plasma (ICP)-atomic emission spectrometry (AES) using an Ultima 2 instrument.

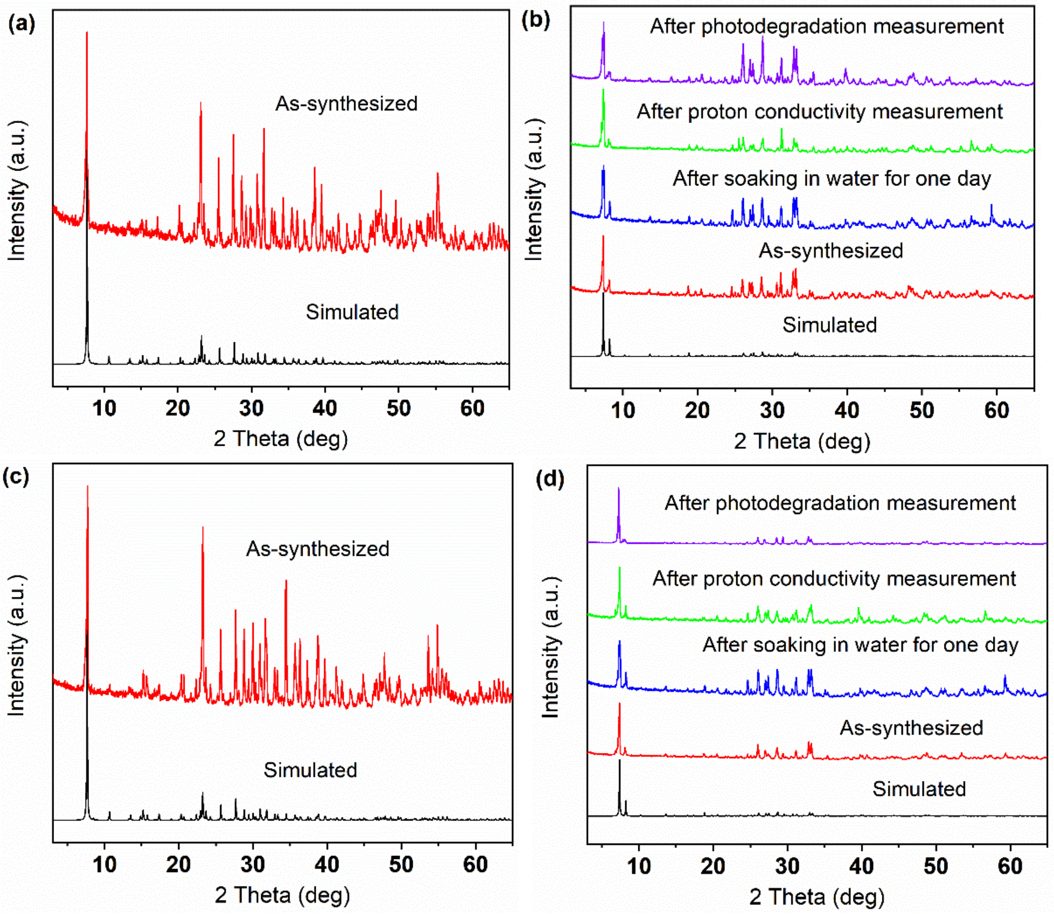

Figure S5. Simulated and observed PXRD patterns of compounds **1** (a), **2** (b), **3** (c), and **4** (d).

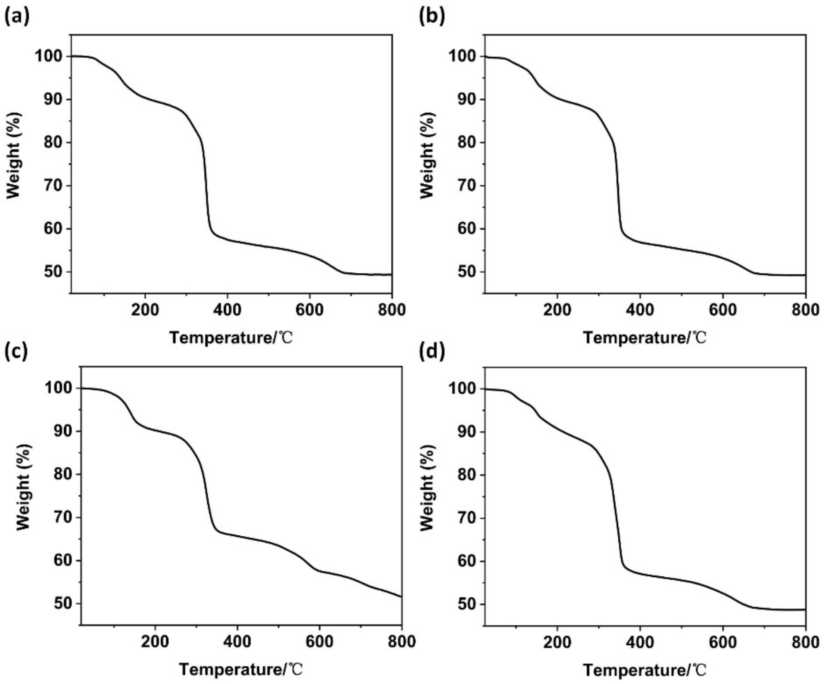

Figure S6. Thermogravimetric curves for compounds **1** (a), **2** (b), **3** (c), and **4** (d).

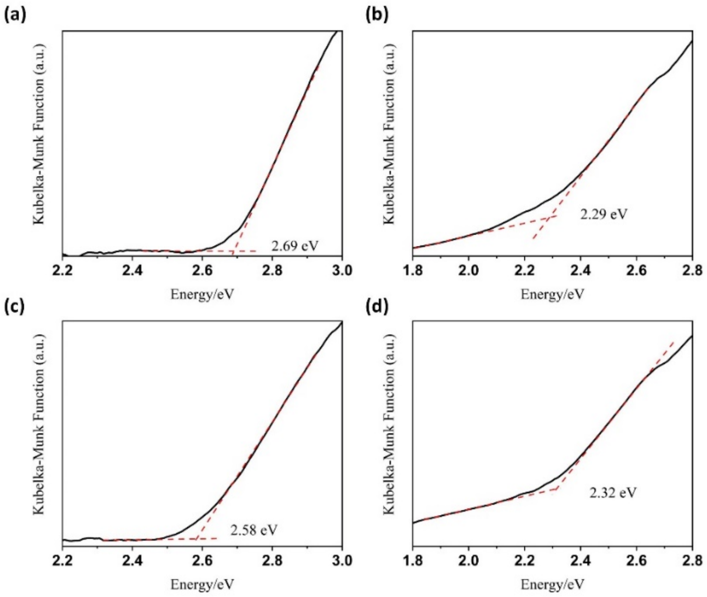

Figure S7. Solid-state UV-Vis absorption spectra of compounds **1** (a), **2** (b), **3** (c), and **4** (d).

Table S2. The bond valence sum calculations for compounds **2** and **4**.

Atom	BVS of **2**	BVS of **4**	Valence
Sb(1)	3.01	3.03	+3
Sb(2)	3.04	3.01	+3
Sb(3)	3.03	3.11	+3
Sb(4)	5.12	5.05	+5
Fe(1)	3.14	3.10	+3
Fe(2)	3.09	3.12	+3
Fe(3)	2.87	2.98	+3
Fe(4)	1.39	1.21	+2
Fe(5)	2.22	2.36	+2
Co(1)	1.94	-	+2
Co(2)	1.85	-	+2
Ni(1)	-	1.85	+2
Ni(2)	-	1.97	+2

Photodegradation of MB measurements

500 ppm standard solution of methylene blue was prepared and diluted to 30, 25, 20, 15, 10, 5, 2 and 1 ppm standard solutions. The absorbance of different concentrations of the standard solutions was measured at the maximum absorption wavelength of 667 nm, and a standard curve of concentration *C* versus absorbance *A* was fitted with concentration *C* (ppm) as the horizontal coordinate and absorbance *A* as the vertical coordinate. Then, 100 mg of the sample was prepared to be ground into powder, which was placed into a photocatalytic reactor, and then 100 mL of 30 ppm methylene blue solution was added, and the solution was left to stir in dark for 0.5 hour. After the adsorption-desorption equilibrium was reached, 4 mL of the masterbatch was taken with a 0.22 um filtering needle and recorded as C_0, and then the sample was analyzed in the PLS-136SXE300UV

Xenon lamp (Beijing Porphyry Science and Technology Co., Ltd.) equipped with a wavelength filter of $\lambda \geq 420$ nm. The photocatalytic degradation experiment was carried out in 4 mL of the mother solution as C_0, and then the absorbance of the sample was measured spectrophotometrically with a 0.22 um filter tip at 1 hour intervals to calculate the concentration of MB solution as C_t. The concentration of methylene blue solution was calculated by using the formula $D = (C_0 - C_t)/C_0 \cdot 100\%$, and the degradation rate was calculated, where D is the degradation rate, C_0 is the initial concentration of the sample after adsorption-desorption equilibrium and C_t is the concentration of MB solution at different time of reaction. A magnetic stirrer at 450 r/min was used throughout the degradation experiments to maintain a homogeneous dispersion of the reactants in the experiments, and an air-cooling system was used to maintain the reaction temperature at 25 °C.

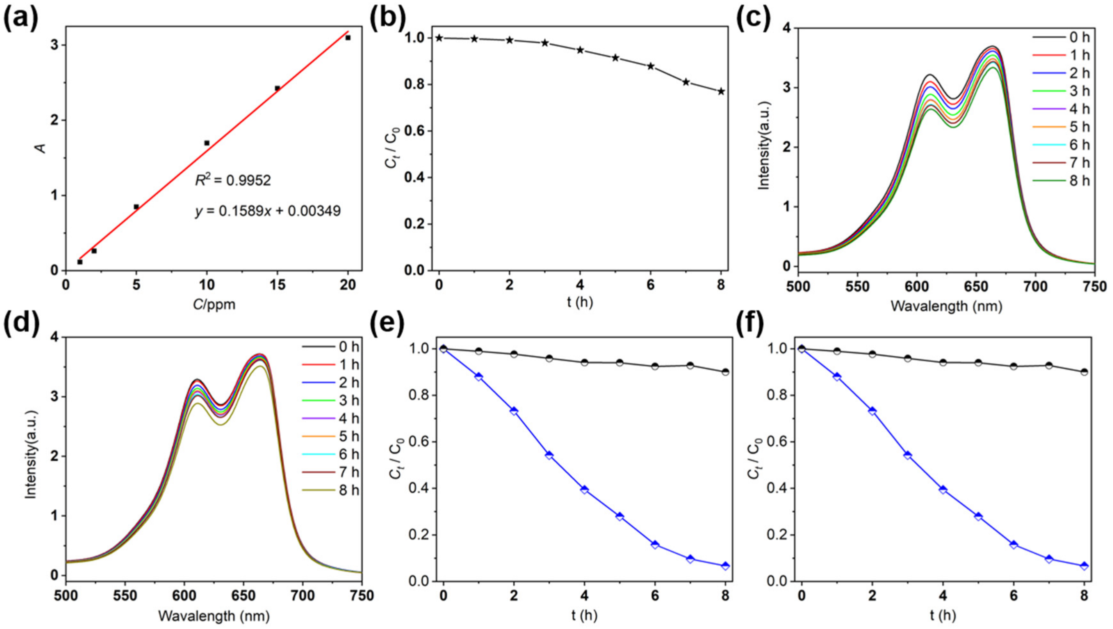

Figure S8. (a) Linearity of absorbance (A) versus standard concentration (C) of MB solution. (b) Curve of C_t/C_0 of MB solution with time (t) in the absence of photocatalyst. Liquid UV-visible absorption spectra of MB solution containing compound **2** (c) and compound **4** (d) under dark conditions at different time. C_t/C_0 curves of MB solution for compounds **2** (e) and **4** (f) as a function of time (t) under dark (black line) or light (blue line) conditions.

Proton conduction measurements

80 mg of compounds **2** and **4** were weighed and ground into powders which were pressed into cylinders with the diameter of 0.50 cm and the height of 1.68 cm (**2**) and 1.71 cm (**4**), respectively, using a mold for proton conduction test. The proton conduction tests were carried out by connecting both ends of the sample to the electrode ends of an impedance analyzer (zennium/IM6) at an AC signal frequency of 1 HZ-10 MHZ and a voltage of 50 mv. The test temperature and relative humidity were controlled by STIK Crop. CIHI-150B constant temperature and humidity chamber. During the test, each temperature condition was maintained for 1 hour. The proton conductivity can be obtained directly from the Nyquist impedance diagram by the formula $\sigma = l/SR$, where l is the length of the sample,

i.e., the height of the cylinder in centimeters, S is the cross-sectional area of the sample, i.e., the bottom area of the cylinder in square centimeters, and R is the electrical resistance of the sample in Ω. The equation for the material activation energy (E_a) value is: $\sigma T = A\ \exp(-E_a/(K_B T))$, σ is the ionic conductivity, A is the prefactor, K_B is the Boltzmann constant, and T is the temperature (K). The measured impedance data were fitted by an equivalent circuit in the ZView software to obtain the resistance value of the sample.

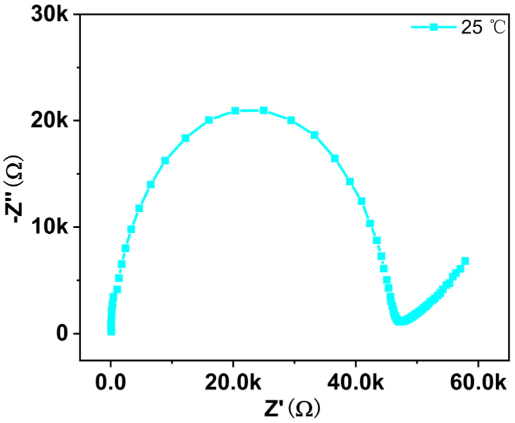

Figure S9. The Nyquist plot from AC impedance data of **2** at 98% RH and 25 °C.

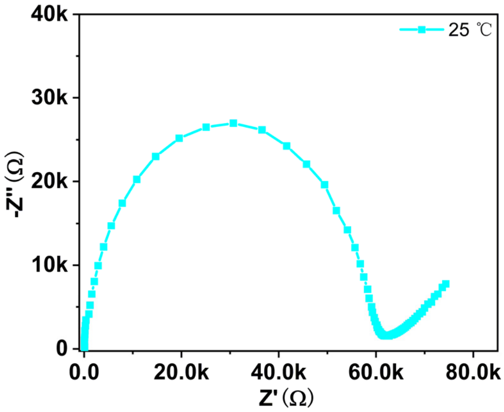

Figure S10. The Nyquist plot from AC impedance data of **4** at 98% RH and 25 °C.

Table S3. Proton conductivity (σ) values of **2** and **4** under 98% RH at different temperatures.

Compound **2**							
T/°C	25	35	45	55	70	75	85
σ (S·cm^{-1})	1.81×10^{-5}	6.45×10^{-5}	1.03×10^{-4}	1.17×10^{-4}	1.96×10^{-4}	2.29×10^{-4}	3.84×10^{-4}
Compound **4**							
T/°C	25	35	45	55	65	75	85
σ (S·cm^{-1})	1.39×10^{-5}	4.91×10^{-5}	8.58×10^{-5}	1.06×10^{-4}	1.2×10^{-4}	1.46×10^{-4}	2.12×10^{-4}

Magnetic measurements

Magnetic measurements were measured on a commercial quantum designed physical property measurement system (PPMS). Ground samples of the two example compounds were weighed into powdered samples of 16.02 mg (**2**) and 11.51 mg (**4**), respectively, and the powdered samples were then tested by placing them in a gel capsule sample holder suspended in a plastic pipette, respectively. The magnetization was tested at an applied field strength of 1000 Oe and in the temperature range of 2 ~ 300 K.

EDS

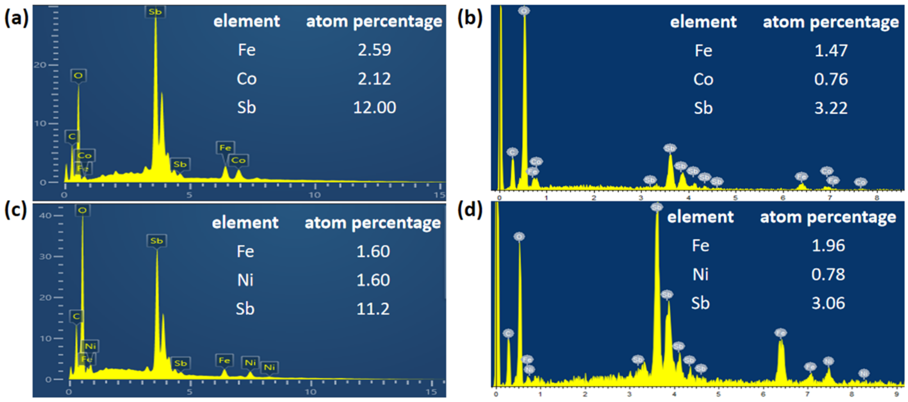

Figure S11. EDS results of compounds **1** (a), **2** (b), **3** (c), and **4** (d).

Table S4. Crystallographic data and refinement details for **1-4**.

Compound	1	2	3	4
Empirical formula	$C_{26}H_{59}NO_{66}Fe_2Co_2Sb_{12}$	$C_{24}H_{43.67}O_{59}Fe_5Co_{2.5}Sb_{6.3}$	$C_{26}H_{59}NO_{66}Fe_2Ni_2Sb_{12}$	$C_{24}H_{46}O_{60}Fe_{5.25}Ni_{2.25}Sb_{6.25}$
Formula weight	3132.30	2473.81	3131.86	2480.85
Temperature/K	297(2)	297(2)	297(2)	297(2)
Crystal system	hexagonal	monoclinic	hexagonal	monoclinic
Space group	$P6_322$	$C2/c$	$P6_322$	$C2/c$
a/Å	13.3439(7)	16.7142(16)	13.3475(9)	16.6795(16)
b/Å	13.3439(7)	21.500(2)	13.3475(9)	21.4879(18)
c/Å	23.9137(15)	17.841(2)	23.824(2)	17.8471(17)
α/°	90	90	90	90
β/°	90	113.938(12)	90	113.721(11)
γ/°	120	90	120	90
V/ Å^3	3687.6	5859.8	3675.8	5856.1
Z	2	4	2	4
D_c/ Mg·m^{-3}	2.821	2.804	2.830	2.814
μ/mm^{-1}	5.258	4.889	5.336	4.934
F(000)	2936.0	4720.0	2940.0	4753.0
R_{int}	0.0469	0.0894	0.0857	0.0640
GOF	1.098	1.053	1.126	1.056

[a]R_1, [b]wR_2 [$I > 2\sigma(I)$]	R_1 = 0.0252, wR_2 = 0.0467	R_1 = 0.0556, wR_2 = 0.1245	R_1 = 0.0372, wR_2 = 0.0588	R_1 = 0.0514, wR_2 = 0.1126
[a]R_1, [b]wR_2 (all data)	R_1 = 0.0333, wR_2 = 0.0503	R_1 = 0.0860, wR_2 = 0.1424	R_1 = 0.0728, wR_2 = 0.0709	R_1 = 0.0812, wR_2 = 0.1310
CCDC number	2313706	2313704	2313703	2313708

[a]$R_1 = \sum \| F_o | - | F_c \| / \sum | F_o |$. [b]$wR_2 = [\sum w(F_o^2 F_c^2)^2 / \sum w(F_o^2)^2]^{1/2}$

Table S5. Selected bond lengths (Å) and angles (°) of compounds **1-4**.

Compound **1**			
Sb(1)-O(1)	2.214(4)	Sb(2)-O(10)	1.978(3)
Sb(1)-O(3)	1.970(4)	Fe(1)-O(10)#3	2.012(4)
Sb(1)-O(10)	2.011(4)	Fe(1)-O(10)#4	2.012(4)
Sb(1)-O(11)	2.122(3)	Fe(1)-O(10)#2	2.012(4)
Sb(2)-O(4)#1	2.258(4)	Fe(1)-O(11)	2.087(4)
Sb(2)-O(6)#1	1.992(4)	Fe(1)-O(11)#1	2.087(4)
Sb(2)-O(9)	2.1192(6)	Fe(1)-O(11)#5	2.087(4)
O(3)-Sb(1)-O(1)	76.82(15)	O(10)#3-Fe(1)-O(10)#4	99.74(13)
O(3)-Sb(1)-O(10)	97.69(15)	O(10)#3-Fe(1)-O(10)#2	99.74(13)
O(3)-Sb(1)-O(11)	79.40(14)	O(10)#4-Fe(1)-O(10)#2	99.74(13)
O(10)-Sb(1)-O(1)	83.00(14)	O(10)#3-Fe(1)-O(11)	96.92(11)
O(10)-Sb(1)-O(11)	76.79(14)	O(10)#4-Fe(1)-O(11)#1	163.34(11)
O(11)-Sb(1)-O(1)	146.24(11)	O(10)#2-Fe(1)-O(11)#1	96.91(11)
O(6)#1-Sb(2)-O(4)#1	75.87(16)	O(10)#3-Fe(1)-O(11)#5	163.34(11)
O(6)#1-Sb(2)-O(9)	80.48(12)	O(10)#3-Fe(1)-O(11)#1	77.55(12)
O(9)-Sb(2)-O(4)#1	148.24(15)	O(10)#2-Fe(1)-O(11)	163.34(11)
O(10)-Sb(2)-O(4)#1	78.82(15)	O(10)#4-Fe(1)-O(11)#5	96.91(11)
O(10)-Sb(2)-O(6)#1	97.06(16)	O(10)#4-Fe(1)-O(11)	77.55(13)
O(10)-Sb(2)-O(9)	84.10(18)	O(10)#2-Fe(1)-O(11)#5	77.54(12)

Symmetry code: #1 2-*y*,1+*x*-*y*,+*z*; #2 2-*y*,2-*x*,3/2-*z*; #3 1+*y*-*x*,+*y*,3/2-*z*; #4 +*x*,1+*x*-*y*,3/2-*z*; #5 1+*y*-*x*,2-*x*,+*z*.

Compound **2**			
Sb(1)-O(1)	2.316(8)	Fe(2)-O(21)	1.985(7)
Sb(1)-O(3)	2.028(8)	Fe(3)-O(10)#1	2.023(8)
Sb(1)-O(19)	2.103(7)	Fe(3)-O(11)#1	2.065(7)
Sb(1)-O(20)	1.955(6)	Fe(3)-O(16)	2.038(8)
Sb(2)-O(7)	2.352(8)	Fe(3)-O(17)	2.033(9)
Sb(2)-O(9)	2.011(7)	Fe(3)-O(20)	2.013(6)
Sb(2)-O(19)	2.076(7)	Fe(3)-O(22)#1	2.018(7)
Sb(2)-O(21)	1.966(6)	Fe(4)-O(16)#1	2.190(8)
Sb(3)-O(13)	2.281(10)	Fe(4)-O(16)	2.190(8)
Sb(3)-O(15)	2.010(7)	Fe(4)-O(22)#1	2.309(11)

Sb(3)-O(19)	2.107(7)	Fe(4)-O(22)	2.309(11)
Sb(3)-O(22)	1.976(6)	Fe(5)-O(1)#1	2.215(9)
Sb(4)-O(20)	2.001(9)	Fe(5)-O(10)	2.079(8)
Sb(4)-O(20)#1	2.001(9)	Fe(5)-O(17)#1	2.237(11)
Sb(4)-O(21)#1	1.95(3)	Fe(5)-O(26)	1.74(2)
Sb(4)-O(21)	1.95(3)	Fe(5)-O(27)	2.163(19)
Sb(4)-O(22)	2.06(3)	Fe(5)-O(28)	2.04(2)
Sb(4)-O(22)#1	2.06(3)	Co(1)-O(4)	2.061(7)
Fe(1)-O(20)#1	1.985(7)	Co(1)-O(5)#1	2.192(9)
Fe(1)-O(20)	1.985(7)	Co(1)-O(7)#1	2.161(7)
Fe(1)-O(21)#1	2.07(4)	Co(1)-O(23)	2.096(10)
Fe(1)-O(21)	2.07(4)	Co(1)-O(24)	2.102(9)
Fe(1)-O(22)#1	1.95(3)	Co(1)-O(25)	2.067(8)
Fe(1)-O(22)	1.95(3)	Co(2)-O(8)#2	2.406(14)
Fe(2)-O(4)	1.978(8)	Co(2)-O(14)	2.042(14)
Fe(2)-O(4)#1	1.978(8)	Co(2)-O(28B)	2.01(4)
Fe(2)-O(5)#1	2.057(7)	Co(2)-O(28C)#1	1.84(6)
Fe(2)-O(5)	2.057(7)	Co(2)-O(28C)	1.67(6)
Fe(2)-O(21)#1	1.985(7)	Co(2)-O(29)	2.248(19)
O(3)-Sb(1)-O(1)	74.0(3)	O(21)-Fe(2)-O(21)#1	79.5(4)
O(3)-Sb(1)-O(19)	78.2(3)	O(10)#1-Fe(3)-O(11)#1	78.1(3)
O(19)-Sb(1)-O(1)	149.1(3)	O(10)#1-Fe(3)-O(16)	155.2(3)
O(20)-Sb(1)-O(1)	84.3(3)	O(10)#1-Fe(3)-O(17)	84.0(4)
O(20)-Sb(1)-O(3)	99.0(3)	O(16)-Fe(3)-O(11)#1	91.3(3)
O(20)-Sb(1)-O(19)	87.0(2)	O(17)-Fe(3)-O(11)#1	107.5(3)
O(9)-Sb(2)-O(7)	74.2(3)	O(17)-Fe(3)-O(16)	77.8(4)
O(9)-Sb(2)-O(19)	79.6(3)	O(20)-Fe(3)-O(10)#1	91.5(3)
O(19)-Sb(2)-O(7)	149.8(3)	O(20)-Fe(3)-O(11)#1	159.5(3)
O(21)-Sb(2)-O(7)	81.9(3)	O(20)-Fe(3)-O(16)	104.8(3)
O(21)-Sb(2)-O(9)	95.4(3)	O(20)-Fe(3)-O(17)	88.6(3)
O(21)-Sb(2)-O(19)	86.1(3)	O(22)#1-Fe(3)-O(10)#1	113.7(3)
O(15)-Sb(3)-O(13)	74.2(3)	O(22)#1-Fe(3)-O(11)#1	89.8(3)
O(15)-Sb(3)-O(19)	78.5(3)	O(22)#1-Fe(3)-O(16)	88.4(3)
O(19)-Sb(3)-O(13)	148.8(3)	O(22)#1-Fe(3)-O(17)	157.9(3)
O(22)-Sb(3)-O(13)	83.1(3)	O(22)#1-Fe(3)-O(20)	78.2(3)
O(22)-Sb(3)-O(15)	98.3(3)	O(16)#1-Fe(4)-O(16)	170.3(6)
O(22)-Sb(3)-O(19)	86.1(2)	O(16)#1-Fe(4)-O(22)#1	110.0(4)
O(20)-Sb(4)-O(20)#1	164(3)	O(16)-Fe(4)-O(22)#1	77.8(3)
O(20)-Sb(4)-O(22)#1	77.6(9)	O(16)-Fe(4)-O(22)	110.0 (4)
O(20)#1-Sb(4)-O(22)#1	91.2(11)	O(16)#1-Fe(4)-O(22)	77.8(3)
O(20)#1-Sb(4)-O(22)	77.6(9)	O(22)#1-Fe(4)-O(22)	78.3(4)
O(20)-Sb(4)-O(22)	91.2(11)	O(1)-Fe(5)-O(17)#1	92.1(3)
O(21)-Sb(4)-O(20)	94.4(9)	O(10)-Fe(5)-O(1)#1	89.3(3)

O(21)#1-Sb(4)-O(20)#1	94.4(9)	O(10)-Fe(5)-O(17)#1	77.9(3)
O(21)-Sb(4)-O(20)#1	97.6(9)	O(10)-Fe(5)-O(27)	92.9(6)
O(21)#1-Sb(4)-O(20)	97.6(9)	O(26)-Fe(5)-O(1)#1	90.0(6)
O(21)#1-Sb(4)-O(21)	81.1(17)	O(26)-Fe(5)-O(10)	104.4(7)
O(21)#1-Sb(4)-O(22)#1	94.8(3)	O(26)-Fe(5)-O(17)#1	177.0(7)
O(21)-Sb(4)-O(22)#1	170.5(9)	O(26)-Fe(5)-O(27)	92.7(8)
O(21)-Sb(4)-O(22)	94.8(3)	O(26)-Fe(5)-O(28)	93.1(9)
O(21)#1-Sb(4)-O(22)	170.5(9)	O(27)-Fe(5)-O(1)#1	176.1(7)
O(22)#1-Sb(4)-O(22)	90.4(18)	O(27)-Fe(5)-O(17)#1	85.1(7)
O(20)-Fe(1)-O(20)#1	173(3)	O(28)-Fe(5)-O(1)#1	91.5(7)
O(20)#1-Fe(1)-O(21)	94.3(11)	O(28)-Fe(5)-O(10)	162.5(8)
O(20)-Fe(1)-O(21)	91.3(11)	O(28)-Fe(5)-O(17)#1	84.6(7)
O(20)-Fe(1)-O(21)#1	94.3(11)	O(28)-Fe(5)-O(27)	85.4(9)
O(20)#1-Fe(1)-O(21)#1	91.3(11)	O(4)-Co(1)-O(5)#1	76.4(3)
O(20)#1-Fe(1)-O(22)	94.8(11)	O(4)-Co(1)-O(7)#1	90.2(3)
O(20)-Fe(1)-O(22)	94.9(11)	O(4)-Co(1)-O(23)	97.6(3)
O(20)#1-Fe(1)-O(22)#1	94.8(11)	O(4)-Co(1)-O(24)	91.3(3)
O(20)-Fe(1)-O(22)#1	80.5(9)	O(4)-Co(1)-O(25)	164.9(4)
O(21)#1-Fe(1)-O(21)	75.6(16)	O(7)#1-Co(1)-O(5)#1	85.3(3)
O(22)-Fe(1)-O(21)	94.4(4)	O(23)-Co(1)-O(5)#1	171.8(4)
O(22)#1-Fe(1)-O(21)	166.5(15)	O(23)-Co(1)-O(7)#1	89.2(4)
O(22)#1-Fe(1)-O(21)#1	94.4(4)	O(23)-Co(1)-O(24)	93.4(4)
O(22)-Fe(1)-O(21)#1	166.5(15)	O(24)-Co(1)-O(5)#1	92.4(4)
O(22)#1-Fe(1)-O(22)	97(2)	O(24)-Co(1)-O(7)#1	176.9(4)
O(4)-Fe(2)-O(4)#1	145.7(5)	O(25)-Co(1)-O(5)#1	88.5(4)
O(4)-Fe(2)-O(5)#1	81.4(3)	O(25)-Co(1)-O(7)#1	89.1(3)
O(4)#1-Fe(2)-O(5)#1	79.2(3)	O(25)-Co(1)-O(23)	97.4(4)
O(4)#1-Fe(2)-O(5)	81.4(3)	O(25)-Co(1)-O(24)	88.7(4)
O(4)-Fe(2)-O(5)	79.2(3)	O(14)-Co(2)-O(8)#2	88.4(5)
O(5)-Fe(2)-O(5)#1	110.2(5)	O(14)-Co(2)-O(29)	85.0(6)
O(21)#1-Fe(2)-O(4)	94.8(3)	O(14)-Co(2)-O(28B)	95.4(11)
O(21)-Fe(2)-O(4)	111.7(3)	O(28B)-Co(2)-O(29)	174.3(10)
O(21)#1-Fe(2)-O(4)#1	111.7(3)	O(28C)-Co(2)-O(14)	128(2)
O(21)-Fe(2)-O(4)#1	94.8(3)	O(28C)#1-Co(2)-O(14)	161(2)
O(21)#1-Fe(2)-O(5)#1	86.2(3)	O(28C)-Co(2)-O(28C)#1	51(4)
O(21)-Fe(2)-O(5)#1	161.1(3)	O(28C)-Co(2)-O(29)	126(2)
O(21)#1-Fe(2)-O(5)	161.1(3)	O(28C)#1-Co(2)-O(29)	84(2)
O(21)-Fe(2)-O(5)	86.2(3)		

Symmetry code: #1 1-*x*,+*y*,1/2-*z*; #2 3/2-*x*,-1/2+*y*,1/2-*z*.

Compound **3**

Sb(1)-O(1)	2.212(5)	Sb(2)-O(10)	1.982(5)
Sb(1)-O(3)	1.976(6)	Fe(1)-O(10)#3	2.012(6)
Sb(1)-O(10)	2.005(6)	Fe(1)-O(10)#2	2.012(6)

Sb(1)-O(11)	2.119(4)	Fe(1)-O(10)#4	2.012(6)
Sb(2)-O(4)#1	2.252(12)	Fe(1)-O(11)	2.088(6)
Sb(2)-O(6)#1	1.993(6)	Fe(1)-O(11)#1	2.088(6)
Sb(2)-O(9)	2.1205(10)	Fe(1)-O(11)#5	2.088(6)
O(3)-Sb(1)-O(1)	76.8(2)	O(10)#4-Fe(1)-O(10)#3	99.62(19)
O(3)-Sb(1)-O(10)	97.9(2)	O(10)#4-Fe(1)-O(10)#2	99.62(19)
O(3)-Sb(1)-O(11)	79.3(2)	O(10)#3-Fe(1)-O(10)#2	99.62(19)
O(10)-Sb(1)-O(1)	83.5(2)	O(10)#4-Fe(1)-O(11)#1	163.43(17)
O(10)-Sb(1)-O(11)	76.8(2)	O(10)#2-Fe(1)-O(11)	96.96(17)
O(11)-Sb(1)-O(1)	146.45(17)	O(10)#3-Fe(1)-O(11)#5	77.38(18)
O(6)#1-Sb(2)-O(4)#1	75.9(2)	O(10)#3-Fe(1)-O(11)#1	96.96(18)
O(6)#1-Sb(2)-O(9)	80.53(16)	O(10)#2-Fe(1)-O(11)#5	163.43(17)
O(9)-Sb(2)-O(4)#1	149.0(3)	O(11)-Fe(1)-O(11)#1	86.75(19)
O(10)-Sb(2)-O(4)#1	79.3(2)	O(11)#1-Fe(1)-O(11)#5	86.75(19)
O(10)-Sb(2)-O(6)#1	97.3(2)	O(11)#5-Fe(1)-O(11)	86.75(19)
O(10)-Sb(2)-O(9)	84.1(3)	O(10)#2-Fe(1)-O(11)#1	77.38(18)
O(10)#4-Fe(1)-O(11)	77.38(19)	O(10)#4-Fe(1)-O(11)#5	96.96(18)
O(10)#3-Fe(1)-O(11)	163.43(17)		

Symmetry code: #1 *-y, +x-y, +z*; #2 *-y,-x*,1/2-*z*; #3 *+x,+x-y*,1/2-*z*; #4 *+y-x,+y*,1/2-*z*; #5 *+y-x, -x, +z*.

Compound **4**

Sb(1)-O(1)	2.338(7)	Fe(2)-O(5)	2.070(6)
Sb(1)-O(3)	2.023(6)	Fe(2)-O(21)	1.965(5)
Sb(1)-O(19)	2.077(6)	Fe(2)-O(21)#1	1.966(5)
Sb(1)-O(20)	1.962(5)	Fe(3)-O(10)#1	2.010(7)
Sb(2)-O(7)	2.356(6)	Fe(3)-O(11)#1	2.067(6)
Sb(2)-O(9)	2.012(6)	Fe(3)-O(16)	2.056(7)
Sb(2)-O(19)	2.094(6)	Fe(3)-O(17)	2.050(6)
Sb(2)-O(21)	1.962(5)	Fe(3)-O(20)	1.978(5)
Sb(3)-O(13)	2.237(7)	Fe(3)-O(22)#1	1.959(5)
Sb(3)-O(15)	2.009(6)	Fe(4)-O(16)#1	2.219(7)
Sb(3)-O(19)	2.108(5)	Fe(4)-O(16)	2.219(7)
Sb(3)-O(22)	1.966(5)	Fe(5)-O(1)#1	2.151(7)
Sb(4)-O(20)#1	1.991(9)	Fe(5)-O(10)	2.074(6)
Sb(4)-O(20)	1.991(9)	Fe(5)-O(17)#1	2.164(7)
Sb(4)-O(21)#1	1.98(4)	Fe(5)-O(26)	1.951(13)
Sb(4)-O(21)	1.98(4)	Fe(5)-O(27)	2.140(11)
Sb(4)-O(22)	2.05(4)	Fe(5)-O(28)	2.042(11)
Sb(4)-O(22)#1	2.05(4)	Ni(1)-O(4)	2.049(6)
Fe(1)-O(20)#1	1.983(6)	Ni(1)-O(5)#1	2.160(7)
Fe(1)-O(20)	1.983(6)	Ni(1)-O(7)#1	2.139(6)
Fe(1)-O(21)#1	2.03(3)	Ni(1)-O(23)	2.087(8)
Fe(1)-O(21)	2.02(3)	Ni(1)-O(24)	2.076(8)

Fe(1)-O(22)#1	2.00(3)	Ni(1)-O(25)	2.036(8)
Fe(1)-O(22)	2.00(3)	Ni(2)-O(14)	1.999(19)
Fe(2)-O(4)#1	1.976(6)	Ni(2)-O(28B)	1.99(4)
Fe(2)-O(4)	1.976(6)	Ni(2)-O(29)	2.274(19)
Fe(2)-O(5)#1	2.070(6)		
O(3)-Sb(1)-O(1)	73.9(2)	O(21)#1-Fe(2)-O(4)#1	111.4(2)
O(3)-Sb(1)-O(19)	79.3(2)	O(21)#1-Fe(2)-O(4)	94.3(2)
O(19)-Sb(1)-O(1)	149.5(2)	O(21)#1-Fe(2)-O(5)#1	87.2(2)
O(20)-Sb(1)-O(1)	82.9(2)	O(21)-Fe(2)-O(5)#1	162.3(2)
O(20)-Sb(1)-O(3)	99.2(2)	O(21)#1-Fe(2)-O(5)	162.2(2)
O(20)-Sb(1)-O(19)	87.4(2)	O(21)-Fe(2)-O(5)	87.2(2)
O(9)-Sb(2)-O(7)	73.9(2)	O(21)-Fe(2)-O(21)#1	80.0(3)
O(9)-Sb(2)-O(19)	80.3(2)	O(10)#1-Fe(3)-O(11)#1	78.3(2)
O(19)-Sb(2)-O(7)	150.3(2)	O(10)#1-Fe(3)-O(16)	151.1(3)
O(21)-Sb(2)-O(7)	82.4(2)	O(10)#1-Fe(3)-O(17)	82.3(3)
O(21)-Sb(2)-O(9)	95.6(2)	O(16)-Fe(3)-O(11)#1	89.3(2)
O(21)-Sb(2)-O(19)	85.9(2)	O(17)-Fe(3)-O(11)#1	106.1(3)
O(15)-Sb(3)-O(13)	74.9(3)	O(17)-Fe(3)-O(16)	76.2(3)
O(15)-Sb(3)-O(19)	77.6(2)	O(20)-Fe(3)-O(10)#1	93.5(2)
O(19)-Sb(3)-O(13)	149.5(3)	O(20)-Fe(3)-O(11)#1	162.6(2)
O(22)-Sb(3)-O(13)	85.0(3)	O(20)-Fe(3)-O(16)	104.6(2)
O(22)-Sb(3)-O(15)	98.3(2)	O(20)-Fe(3)-O(17)	87.7(2)
O(22)-Sb(3)-O(19)	86.1(2)	O(22)#1-Fe(3)-O(10)#1	115.4(2)
O(20)#1-Sb(4)-O(20)	164(3)	O(22)#1-Fe(3)-O(11)#1	89.7(2)
O(20)-Sb(4)-O(22)#1	77.5(11)	O(22)#1-Fe(3)-O(16)	90.2(2)
O(20)#1-Sb(4)-O(22)#1	91.8(14)	O(22)#1-Fe(3)-O(17)	158.7(2)
O(20)-Sb(4)-O(22)	91.8(14)	O(22)#1-Fe(3)-O(20)	79.9(2)
O(20)#1-Sb(4)-O(22)	77.5(11)	O(16)-Fe(4)-O(16)#1	173.7(16)
O(21)#1-Sb(4)-O(20)	98.2(12)	O(1)#1-Fe(5)-O(17)#1	92.8(3)
O(21)-Sb(4)-O(20)#1	98.2(12)	O(10)-Fe(5)-O(1)#1	88.2(2)
O(21)#1-Sb(4)-O(20)#1	93.9(11)	O(10)-Fe(5)-O(17)#1	78.1(2)
O(21)-Sb(4)-O(20)	93.9(11)	O(10)-Fe(5)-O(27)	92.5(3)
O(21)-Sb(4)-O(21)#1	80(2)	O(26)-Fe(5)-O(1)#1	87.1(4)
O(21)-Sb(4)-O(22)#1	168.3(14)	O(26)-Fe(5)-O(10)	104.0(4)
O(21)#1-Sb(4)-O(22)#1	93.8(2)	O(26)-Fe(5)-O(17)#1	177.8(4)
O(21)-Sb(4)-O(22)	93.8(2)	O(26)-Fe(5)-O(27)	94.5(5)
O(21)#1-Sb(4)-O(22)	168.3(14)	O(26)-Fe(5)-O(28)	93.3(5)
O(22)-Sb(4)-O(22)#1	94(2)	O(27)-Fe(5)-O(1)#1	178.1(4)
O(20)#1-Fe(1)-O(20)	168(2)	O(27)-Fe(5)-O(17)#1	85.6(4)
O(20)-Fe(1)-O(21)	92.6(8)	O(28)-Fe(5)-O(1)#1	90.7(4)
O(20)#1-Fe(1)-O(21)	96.8(9)	O(28)-Fe(5)-O(10)	162.6(4)
O(20)-Fe(1)-O(21)#1	96.8(9)	O(28)-Fe(5)-O(17)#1	84.5(4)
O(20)#1-Fe(1)-O(21)#1	92.5(8)	O(28)-Fe(5)-O(27)	88.0(5)

O(20)-Fe(1)-O(22)	93.3(9)	O(4)-Ni(1)-O(5)#1	77.8(2)
O(20)#1-Fe(1)-O(22)	78.6(7)	O(4)-Ni(1)-O(23)	96.1(3)
O(20)#1-Fe(1)-O(22)#1	93.3(9)	O(4)-Ni(1)-O(7)#1	89.0(2)
O(20)-Fe(1)-O(22)#1	78.6(7)	O(4)-Ni(1)-O(24)	92.6(3)
O(21)-Fe(1)-O(21)#1	77.2(14)	O(7)#1-Ni(1)-O(5)#1	87.4(2)
O(22)#1-Fe(1)-O(21)#1	93.6(3)	O(23)-Ni(1)-O(5)#1	172.6(3)
O(22)-Fe(1)-O(21)#1	166.6(12)	O(23)-Ni(1)-O(7)#1	88.4(3)
O(22)-Fe(1)-O(21)	93.6(3)	O(24)-Ni(1)-O(5)#1	90.3(3)
O(22)#1-Fe(1)-O(21)	166.6(12)	O(24)-Ni(1)-O(7)#1	176.9(3)
O(22)-Fe(1)-O(22)#1	97.0(17)	O(24)-Ni(1)-O(23)	94.1(3)
O(4)-Fe(2)-O(4)#1	146.7(4)	O(25)-Ni(1)-O(4)	168.9(3)
O(4)-Fe(2)-O(5)#1	81.6(2)	O(25)-Ni(1)-O(5)#1	91.3(3)
O(4)-Fe(2)-O(5)	78.9(2)	O(25)-Ni(1)-O(7)#1	88.3(3)
O(4)#1-Fe(2)-O(5)	81.6(2)	O(25)-Ni(1)-O(23)	94.6(4)
O(4)#1-Fe(2)-O(5)#1	78.9(2)	O(25)-Ni(1)-O(24)	89.6(3)
O(5)#1-Fe(2)-O(5)	107.7(3)	O(14)-Ni(2)-O(28B)	93.4(13)
O(21)-Fe(2)-O(4)#1	94.3(2)	O(14)-Ni(2)-O(29)	85.9(7)
O(21)-Fe(2)-O(4)	111.4(2)	O(28B)-Ni(2)-O(29)	171.8(12)

Symmetry code: #1 1-*x*,+*y*,1/2-*z*.
